# A Model for Sigma Factor Competition in Bacterial Cells

**DOI:** 10.1371/journal.pcbi.1003845

**Published:** 2014-10-09

**Authors:** Marco Mauri, Stefan Klumpp

**Affiliations:** Max Planck Institute of Colloids and Interfaces, Potsdam, Germany; University of Illinois at Urbana-Champaign, United States of America

## Abstract

Sigma factors control global switches of the genetic expression program in bacteria. Different sigma factors compete for binding to a limited pool of RNA polymerase (RNAP) core enzymes, providing a mechanism for cross-talk between genes or gene classes via the sharing of expression machinery. To analyze the contribution of sigma factor competition to global changes in gene expression, we develop a theoretical model that describes binding between sigma factors and core RNAP, transcription, non-specific binding to DNA and the modulation of the availability of the molecular components. The model is validated by comparison with *in vitro* competition experiments, with which excellent agreement is found. Transcription is affected via the modulation of the concentrations of the different types of holoenzymes, so saturated promoters are only weakly affected by sigma factor competition. However, in case of overlapping promoters or promoters recognized by two types of sigma factors, we find that even saturated promoters are strongly affected. Active transcription effectively lowers the affinity between the sigma factor driving it and the core RNAP, resulting in complex cross-talk effects. Sigma factor competition is not strongly affected by non-specific binding of core RNAPs, sigma factors and holoenzymes to DNA. Finally, we analyze the role of increased core RNAP availability upon the shut-down of ribosomal RNA transcription during the stringent response. We find that passive up-regulation of alternative sigma-dependent transcription is not only possible, but also displays hypersensitivity based on the sigma factor competition. Our theoretical analysis thus provides support for a significant role of passive control during that global switch of the gene expression program.

## Author Summary

Bacteria respond to changing environmental conditions by switching the global pattern of expressed genes. A key mechanism for global switches of the transcriptional program depends on alternative sigma factors that bind the RNA polymerase core enzyme and direct it towards the appropriate stress response genes. Competition of different sigma factors for a limited amount of RNA polymerase is believed to play a central role in this global switch. Here, a theoretical approach is used towards a quantitative understanding of sigma factor competition and its effects on gene expression. The model is used to quantitatively describe *in vitro* competition assays and to address the question of indirect or passive control in the stringent response upon amino acids starvation. We show that sigma factor competition provides a mechanism for a passive up-regulation of the stress specific sigma-driven genes due to the increased availability of RNA polymerase in the stringent response. Moreover, we find that active separation of sigma factor from the RNA polymerase during early transcript elongation weakens the sigma factor-RNA polymerase equilibrium constant, raising the question of how their *in vitro* measure is relevant in the cell.

## Introduction

During recent years, much effort has been made towards the quantitative characterization of gene regulation and regulatory networks [1]–[5]. In a reductionist spirit, gene regulation has usually been studied one gene at a time. Even in genome wide studies to characterize regulons, the focus has been on the direct effects of, for example, a specific transcription factor. However, it has become increasingly clear that genes are coupled both to each other and to the state of the cell as a whole. Specific cross-talk has been demonstrated in a number of systems, for example for small regulatory RNAs [6], proteases [7] and transcription factor binding [8]. In addition, genes are generically coupled to each other through the transcription and translation machinery they share [9]–[12]. At the level of translation, the mRNA transcripts of different genes are in competition for a limiting pool of ribosomes. In *Escherichia coli* this competition is indicated by the re-distribution of ribosomes between protein classes upon changes in cell growth conditions [10], [13] and by the (transient) down-regulation of translation of unrelated mRNAs upon induction of a gene from a high-copy number plasmid [14].

At the level of transcription, such coupling appears to be weaker, as RNA polymerase core enzyme is available in excess of the numbers needed for transcription [15], [16]. However, sigma factors, which bind core RNAP and which are required for bacterial RNA polymerase to recognize promoters are generally believed to be subject to competition for binding core RNAP [17]. Bacteria typically have several types of sigma factors that are activated during different conditions, recognize different classes of promoters and direct transcription to specific cellular programs [17], [18]. A housekeeping sigma factor (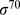 in *E. coli*, 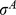 in *B. subtilis*) is required for most transcription during growth, while other sigma factors act as master regulators for stress responses such as heat shock or entry to stationary phase (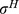 and 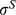, respectively in *E. coli*) or for developmental programs such as growth of flagella (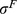 in *E. coli*) and sporulation (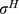, 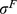, 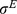, 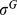, 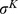 in *B. subtilis*). In addition some phages carry genes for sigma factors that direct transcription to phage genes [19], [20]. The switch between the different transcriptional programs is driven by the modulation of the availability of sigma factors through regulation of their transcription and translation, regulated proteolysis and sequestering by anti-sigma factors [17], [21]–[23]. When more than one sigma factor is present in the cell at the same time, they are believed to compete for core RNA polymerase. Evidence for sigma factor competition in bacterial cells has come from overexpression experiments modulating the level of sigma factors and from mutants with altered sigma-core dissociation constants [24]–[28]. In addition, sigma factor competition has been demonstrated in *in vitro* transcription assays [19], [20], [26], [29]–[34].

As a result of competition, any increase in activity of one sigma factor indirectly represses binding of other sigma factors to core RNAP and thus transcription of the genes they control. Such passive control has been proposed to contribute to the switch of the global gene expression program [35]. In recent years this scenario was specifically proposed to occur in the so-called stringent response, a stress response to lack of amino acid, and during entry to stationary phase [29], [34]–[37]. In both cases, the stop or down-regulation of transcription of ribosomal RNA represents a major perturbation of the allocation of (core) RNA polymerases to different genes and to different sigma factors. However, previous theoretical analysis of other passive effects has shown that a quantitative analysis is required as many cellular parameters change at the same time and may have opposing effects on the genes of interest, so that their net effect may not be obvious. Specifically for 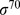 dependent biosynthetic operons, it has been argued that passive effects only play a minor role [15].

In this article, we develop a model for sigma factor competition to quantitatively analyze different situations. Our model is based on and extends previous theoretical work on sigma factor competition by Grigorova *et al.* [38]. We first use a reduced core model to quantitatively analyze *in vitro* competition experiments from the literature [29], [30] and find good agreement between the model and the data. Then we extend the model to include the non-specific DNA binding, which has previously been shown to buffer against passive effect in *σ* ^70^-dependent transcription against passive effects such as an increased RNA polymerase concentration due to the stop of ribosomal RNA transcription. By contrast, we show here that non-specific binding does not buffer alternative *σ*-dependent transcription against such passive effects, supporting a role for passive up-regulation of alternative *σ*-dependent stress response genes [15]. Moreover, we include an explicit description of transcript elongation, which we show to have rather complex effects by modulating the effective sigma-core binding affinity in addition to sequestering RNAP core enzymes. Finally, we apply the model to the increase in the availability of core RNAP during the stringent response and show that passive up-regulation should indeed play an important role for alternative sigma-dependent transcription.

## Results

### Model for sigma factor competition

**Figure 1:**
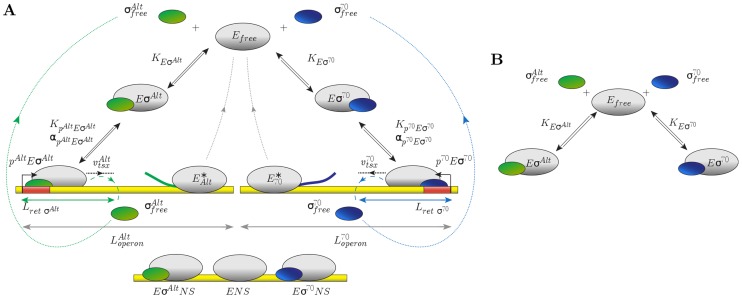
Model for sigma factor competition. (A) Model for sigma factor competition with two types of sigma factors, the housekeeping sigma factor 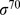 and a generic alternative sigma factor 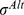: the model describes binding of 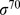 or 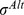 to core RNA polymerase (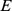) to form holoenzymes (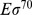 and 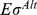) as well as transcription (promoter binding, transcription initiation and elongation) of the cognate genes and non-specific binding of holoenzymes and core RNAPs to DNA. (B) Core model for holoenzyme formation.

To analyze sigma factor competition, we have developed a quasi-steady state model based on earlier work by Grigorova *et al.* [38]. Our model (Figure 1A) describes the interaction between sigma factors and core RNAPs. Core RNAPs (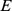) bind to sigma factors (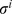, where 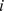 denotes the type of sigma factor) to form holoenzymes (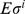). The binding is characterized by a dissociation constant 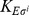. Holoenzymes specifically recognize a cognate class of promoters, where they initiate transcription. After initiation of transcription, the sigma factor is released in a stochastic fashion and the core RNAP transcribes until it reaches a termination sequence. Once set free, the subunits return to the pool of free sigma factors and cores, respectively. This cycle enables the reprogramming of RNAPs by different sigma factors. Holoenzymes and core RNAPs can also bind non-specifically to DNA. In the following we will discuss this model step-by-step, starting with the core model of Figure 1B. A detailed mathematical formulation of the model is given in Methods and in Text S1.

For simplicity, we focus on the case of only two competing sigma factors, the housekeeping sigma factor 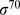, and one type of alternative sigma factor, which we denote by 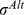, as shown in Figure 1B. This simplification can be interpreted in two ways: it provides a good description of specific stress responses, in which only one specific alternative sigma factor accumulates. Alternatively, it applies also to a general stress response, in which most or all alternative sigma factors are induced simultaneously, if these are lumped together into a single group of alternative sigma factors, assuming that their parameters are rather similar. The competition of sigma factors for core RNAP depends on five parameters: the concentrations of cores and sigma factors and the dissociation constants between them. Unless specified otherwise, we quantify the amounts of the various molecular species by their absolute number in an average cell, taken to have the characteristic volume of 1.32 fL (parameter values are summarized in Table 1, their derivation is discussed in the Text S2).

**Table 1:** Values adopted in the simulations.

Quantity	Assumed Value
Average cell volume	1.32 fL
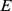 per cell	11400
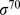 per cell	5700
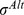 per cell	from 0 to 20000
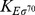 , 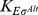	1 nM
Anti - 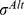 per cell	5000
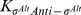	0.01 nM
Anti - 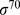 per cell	19000
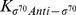	50 nM
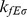	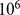 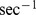 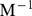
Genome equivalent per cell	3.8
Non-specific binding sites per cell	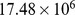
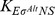 , 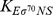 , 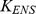	from 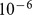 M to 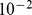 M
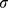 -cognate promoters per cell	200
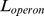	2000 nt
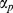	40 min 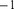
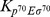 , 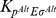 , 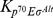	from 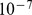 M to 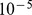 M
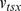	55 nt sec 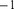
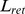	300 nucleotides

For a discussion of the parameters, see Text S2.

**Figure 2:**
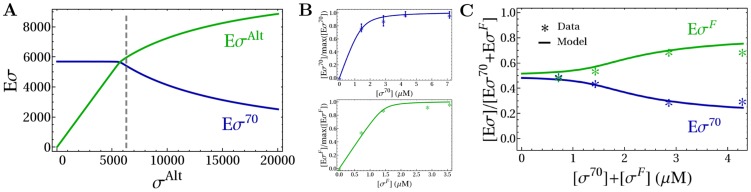
Holoenzyme formation. (A) Number of holoenzymes 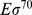 and 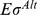 as a function of the copy number of alternative sigma factors. Quantities of all molecular species are expressed as absolute numbers per cell. The gray dashed line represents the onset of the competition, when 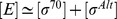. The values of the parameters used in the simulations are summarized in Table 1. (B) Determination of the sigma-core dissociation constants for 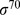 and 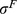 (see Table 2) by fitting the results of binding assays between cores and sigma factors [30], [39]. The number of core-sigma complexes normalized to the maximal number of holoenzymes, 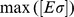. Stars show the experimental data and lines are due to the fit. (C) Comparison of model predictions (lines) with an *in vitro* competition experiment [30] with a fixed amount of core and different equimolar amounts of 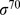 and 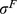 (stars) in the same conditions as in (B). The plot shows the fraction of sigma factors bound in holoenzymes as a function of the total sigma factor concentration, 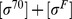.

We consider fixed concentrations of core RNAP and 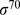, here 11400 and 5700 molecules, respectively, as in a rapidly growing *E. coli* cell, and modulate the concentration of 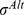. This situation is accessible to *in vitro* experiments and mimics the accumulation of alternative sigma factors during the transition from exponential to stationary phase. First, we study the formation of holoenzymes in the absence of transcription (*i.e.* no DNA present) as in Figure 1B. Figure 2A shows the amounts (number per cell) of the two species of holoenzymes as functions of the number of alternative sigma factors. Both sigma factors are taken to bind to core RNAP with equal dissociation constants of 1 nM. As long as the total concentration of sigma subunits is smaller than that of core RNAPs, there are enough cores to bind all sigma factors. In that case, the number of alternative holoenzymes increases linearly in the number of alternative sigma factor and formation of 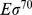 is unaffected by the increasing concentration of 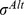, i.e. there is no competition for core RNAP or no cross-talk between the two branches of the system. Competition sets in and the formation of 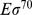 gets reduced by the presence of the alternative sigma factor, when the total concentration of sigma factors exceeds the concentration of cores RNAPs as observed previously [38]. For strong binding between core and sigma, the onset of competition is sharp as in Figure 2A. If the binding is weaker (larger dissociation constant), competition sets in more smoothly. In that case, we define the onset of competition to occur when the presence of alternative sigma decreases the 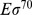 production by 5% with respect to the reference conditions without alternative sigma factors (Equation 9 in Methods). The starting point of the competition defined in this way is indicated by a grey dashed vertical line in Figure 2A and in the following plots. Thus, when the total concentration of sigma factors exceeds the concentration of cores, an increase in availability of alternative sigma factors indirectly down-regulates the production of housekeeping holoenzymes. We note that if housekeeping sigma factor is already in excess of core, any small number of alternative sigma factor will be in competition with 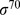. Then the criterion of 5% reduction leads to an additional limiting condition for competition (Equation S1 in Text S1). If the dissociation constants of the two holoenzymes are different, when varying the availability of core RNAP, this criterion can result in a competition in an intermediate range of core concentration, as we will show below.

We next used the model described so far to analyze an *in vitro* competition experiment between 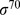 and 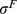. In reference [30], a fixed amount of core RNAP was first mixed with increasing concentrations of either 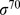 [39] or 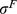 [30] to determine the amount of produced holoenzymes. Fitting this data with our model (Equation 3 in Methods), we determined the dissociation constants between core and sigma subunits (Table 2 and Figure 2B). Then, in a competition assay under the same conditions, different equimolar concentrations of 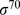 and 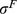 were mixed with a fixed amount of cores to determine the fraction of corresponding holoenzymes produced in the reaction [30]. The latter experimental results are shown as stars in Figure 2C. Using the dissociation constants determined by the fit together with the known concentrations of sigma factors and core RNAPs, we can quantitatively calculate the holoenzyme fractions in the competition experiment with our model. The results are shown as solid lines in Figure 2C and are found to be in good agreement with the experimental data.

**Table 2:** Fit values.

Parameter	Fit value	Reference	Used in Figure
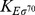	130 nM	[39]	2B, 2C
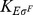	25 nM	[30]	2B, 2C
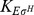	98.2 nM	[29]	3B, 3C
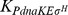	24.5 nM	[29]	3B, 3C
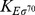	21.1 nM	[32]	3B, 3C

Summary of the fit values that we have used in our binding affinity simulations.

The concentration of a certain species of holoenzyme can be written as a function of the concentration of the holoenzymes of a competing species, their relative dissociation constants, and the total number of sigma factors as
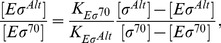
(1)see Methods. As a special case, this equation implies that core RNAPs are equally distributed among different sigma species when these are present in equal amounts and have same affinity for the core. We note that this equation is also valid if more than two species of sigma factors are present. In this case it can be applied to each pair of sigma factors to determine the relative dissociation constants and thus the hierarchy of sigma-core binding. This analysis is shown in Figure S1 for an *in vitro* competition experiment among the seven sigma factors of *E. coli* performed by Maeda *et al.* [31]. Using Equation 1, we find the binding hierarchy shown in Table S1, which differs slightly from the one obtained by Maeda *et al.* from the same data using a fit that assumed a saturation condition.

**Figure 3:**
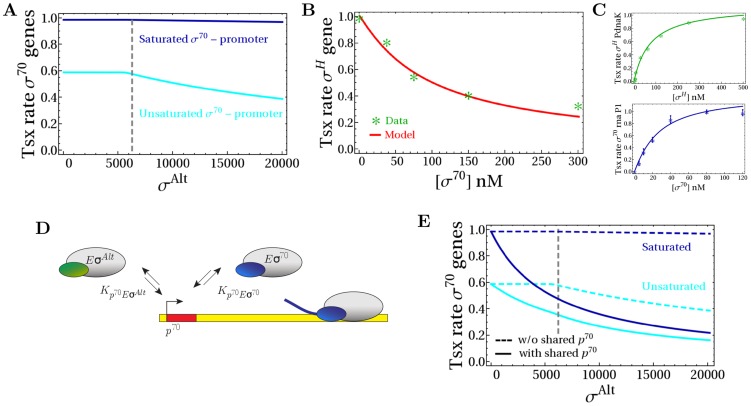
Transcription rate. (A) Normalized transcription rate 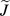 (Equation 12) for a *σ* ^70^-dependent promoter as a function of the number of alternative sigma factors. The numbers of 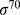 and cores are fixed. The blue line is for a saturated promoter (with 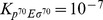 M) and the cyan line for an unsaturated promoter (with 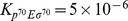 M). (B) Comparison of model predictions (lines) with an *in vitro* competition experiment [29] with a fixed amount of core and *σ^H^* and different amounts of 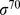 (stars). The plot shows the transcription rate of a *σ^H^*-dependent gene (normalized to the maximal value) as a function of the concentration 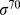. (C) The sigma-core and the holoenzyme-promoter dissociation constants (see Table 2) are determined by fitting the results of transcription rate experiments with a fixed amount of cores in the same conditions as in (B) without competition in the presence of a DNA template containing *σ^H^*- and *σ* ^70^-driven genes [29], [32]. (D) When a *σ* ^70^-dependent promoter also binds another type of holoenzyme or overlaps to another promoter, 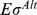 also acts as a repressor of the *σ* ^70^-dependent transcription. (E) Normalized transcription rate of a saturated and unsaturated *σ* ^70^-dependent promoter as a function of the number of 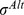 (blue and cyan solid lines with 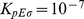 M and 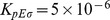 M, respectively). The dashed line show the corresponding results in the absence of repression by promoter sharing or overlapping.

Next, we examine the transcription rates. Each holoenzyme species transcribes a set of cognate genes with a transcription rate that depends on the holoenzyme concentration and on the parameters of the promoter, which is described with a Michaelis-Menten model (see Methods). We assume that only a small number of RNAPs are transcribing at any time, so that the pools of non-transcribing holoenzymes and free subunits are not perturbed by transcription. This assumption should be valid for *in vitro* experiments, but may not hold in the cell; the latter case will be discussed below. Figure 3A shows the transcription rate of the *σ* ^70^-dependent promoter as a function of the increasing amount of 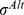, again keeping the concentrations of core RNAP and 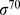 constant. The transcription rate shows a strong dependence on the Michelis constant of the promoter, 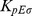 (which corresponds to a holoenzyme-promoter dissociation constant in the limit where binding is equilibrated before transcription is initiated). For unsaturated promoters (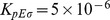 M, cyan line), the transcription rate directly reflects the holoenzyme concentration of Figure 2A: upon the onset of competition, transcription from the 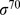 promoter is reduced, as the increasing amount of 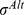 diverts core RNAPs to form alternative holoenzymes. Saturated promoters (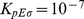 M, blue line) are much less affected by an increasing concentration of 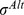. Thus, unsaturated promoters are more sensitive to sigma factor competition than saturated promoters.

The prediction for the transcription rate can be compared to another in vitro competition experiment, this time between 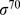 and 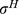 [29]. In this experiment, a DNA template containing the *σ^H^*-dependent PdnaK promoter was mixed with fixed concentrations of RNAPs and 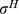 and an increasing concentration of 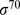. The measured transcription rates are shown in Figure 3B as green stars. To reproduce these observations with our model, we need to determine the required parameters: the sigma-core dissociation constants, 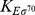, 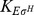, and the holoenzyme-cognate promoter dissociation constant, 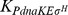. To that end, we fit two experiments [29], [32] done in the same conditions of the mixing assay, but in the presence of a single sigma factor species (using Equations 3 and 12, see Methods). The results of the fits are summarized in Table 2 and in Figure 3C. Once we have all the parameters, we use our model to calculate the transcription rate under conditions of sigma competition. The result is plotted as solid red line in Figure 3B and agrees well with the experimental data. The quantitative agreements between our calculation and experiments provides validation for the modeling approach to sigma factor competition that we use here.

ChIP-chip experiments with different sigma factors have shown that many promoters can bind more than one kind of holoenzyme, even though only one type may successfully initiate the transcription of the gene [40], [41]. In these particular instances the non-transcribing holoenzyme effectively acts as a transcriptional repressor for the gene in addition to competing for core RNAP (Figure 3D and Equation 13 in Methods). The additional function can strongly enhance the negative effect of the alternative sigma factor on *σ* ^70^-driven transcription (Figure 3E). In particular, it also affects saturated promoters that are only weakly affected by sigma factor competition (blue line in Figure 3E). Our findings suggest that competition for shared promoters contributes to the repression of transcription of the associated genes, specifically in the case where these genes are predominantly transcribed by one of the holoenzyme species binding to the promoter. Evidence for such repression was found in a very recent genome-wide study of sigma factor–promoter binding [41] and qualitatively agrees with the picture resulting from our model: most *σ^S^*-dependent genes were found to be down-regulated by knocking out *rpoS* (the genes encoding 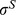). Those *σ^S^*-dependent genes that are up-regulated were found to be genes that are transcribed by both 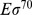 and 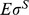 and to which the housekeeping holoenzyme binds more strongly.

Sigma factor availability can be modulated by anti-sigma factors which bind to a cognate sigma factor and thus prevent holoenzyme formation [21], [42]. Figure S2A shows the effect of a fixed number of anti-sigma factors sequestering alternative sigma factors (anti-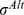). On the one hand, formation of alternative holoenzymes (solid green line) is strongly suppressed as long as the number of anti-sigma factors exceeds the number of sigma factor and sets in rather abruptly as this threshold is crossed. This effect has been described previously and was proposed as a sensitive regulatory element for the design of synthetic gene circuits [43] and as a key ingredient for bistability in the mycobacterial stress response [44]. On the other hand, onset of competition with the housekeeping sigma factor is shifted towards larger numbers of 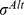 compared to the case without the anti-sigma factor (dashed lines), as binding between sigma and anti-sigma factors effectively reduces the number of 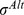 molecules that participate in the competition. However, the results are also dependent on the relative binding strength between the sigma factor on the one hand and the anti-sigma factor or core RNAP on the other hand. This is illustrated in Figure S2B for the case of a large number of anti-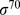 that binds housekeeping sigma factor relatively weakly, as it is the case for Rsd and AsiA [45]. Here, addition of anti-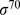 leads to an apparent shift in the onset of competition to lower values of alternative sigma factor (red arrow), even though 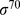 are removed from the competition by the anti-sigma factor. One can see that for small 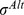, the main effect of the anti-sigma factor is a decrease in 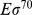 without a concomitant increase in 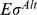. Thus, in this regime, the presence of the alternative sigma factors enhances binding between the housekeeping sigma factor and the cognate anti-sigma factor.

### Modulation of sigma factor competition by non-specific DNA binding

In addition to their specific binding to promoters, holoenzymes as well as core RNAPs can also bind to DNA non-specifically, in an approximately sequence-independent manner [46]. Despite being weak, non-specific binding may have a strong effect because of the great abundance of non-specific binding sites [1], [15]. Non-specific binding of RNAPs to DNA has been proposed to keep weak promoters unsaturated as a prerequisite for the positive control of transcription [38] and to buffer the free RNAP concentration against strong modulation by the stop of transcription of highly expressed genes [15].

**Figure 4:**
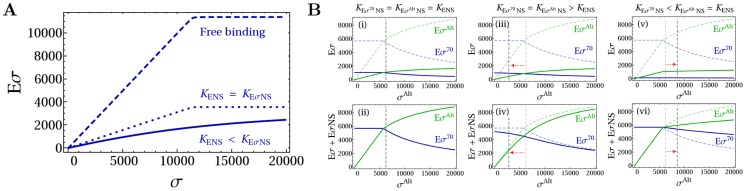
Effect of non-specific binding of holoenzymes and cores to DNA. (A) Formation of holoenzymes in the presence of one type of sigma factor in the absence of DNA (no non-specific binding, dashed line), in the presence of DNA with equal non-specific binding affinities of cores and holoenzymes (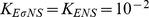 M, dotted line) and with different non-specific binding affinities (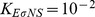 M, 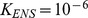 M, solid line). (B) Number of free cytoplasmic holoenzymes 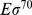 and 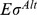 (upper row) and total number of holoenzymes (free and non-specifically bound, 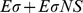, lower row) as functions of the copy number of alternative sigma factors for three different combinations of non-specific binding affinities: in (i) and (ii) all non-specific dissociation constant are equal (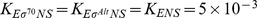 M), in (iii) and (iv) the non-specific dissociation constant for the core is smaller than for the holoenzymes (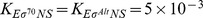 M, 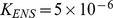 M), in (v) and (vi) the non-specific dissociation constant for the 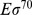 is smaller than for 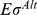 and core (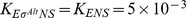 M, 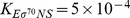 M). The dashed lines in all panels shows the reference case without DNA (no non-specific binding).

In our model, using parameters expected for the situation in the cell (a relatively large non-specific dissociation constant 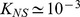
M [1], [15] and a total of 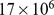 binding sites given by 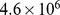 base pairs per genome times 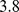 genome equivalents present in a rapidly growing *E. coli* cell), we find that non-specific binding strongly reduces the concentration of free holoenzymes and, thus, specific binding to promoters. In Figure 4A, for only one type of sigma factor, the dashed line shows the reference state without non-specific binding, the dotted and solid lines cases with non-specific binding. If non-specific DNA binding of core RNAPs and holoenzymes are characterized by the same (or approximately the same) dissociation constant (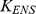 and 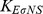, respectively, dotted line in Figure 4A), non-specific DNA binding does not affect sigma-core binding, and the total number of holoenzymes is the same as without non-specific binding. In that case, the concentration of free holoenzymes is simply rescaled with the probability that a holoenzyme is free in the cytoplasm, 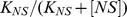 (see Methods), compared to the case without non-specific binding (dotted and dashed line in Figure 4A, respectively). This property is lost when the non-specific dissociation constants are different (solid line). For example, if core RNAP binds to DNA more strongly than holoenzyme, non-specific DNA competes with 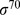 for core binding and thereby reduces the concentration of (both total and free) holoenzymes. From an experimental point of view, dissociation constants for non-specific binding are dependent on ionic conditions, due to the electrostatic nature of non-specific binding, with a stronger dependence for core than for 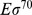 [46]. Under physiological high-salt conditions, 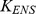 and 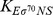 are expected to be rather similar [46], so that sigma-core binding is not affected by the presence of non-specific DNA. However, a difference in the dissociation constants could affect *in vitro* transcription if different experimental conditions are used.

A similar result is obtained for the competition of two sigma factors (see Figure 4B): if the two holoenzymes and core RNAPs have the same binding affinity for non-specific DNA (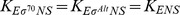, solid lines in panels (i) and (ii)), non-specific binding does not affect sigma factor competition and free concentrations of holoenzymes are obtained by a simple rescaling of the total concentrations of holoenzymes (panel (i)). Under these conditions, both free and non-specifically bound core RNAPs participate in sigma factor competition as shown in panel (ii), where we plot the total number of holoenzymes (free and non-specifically bound). Here, the solid lines (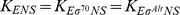) fall on top of the dashed lines, which show the case without non-specific binding. When one of the non-specific dissociation constants is different, however, the rescaling property is lost and the onset of sigma factor competition is shifted, as shown by the red arrows and solid lines in panels (iii)–(vi). In panels (iii) and (iv), 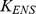 is smaller than 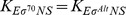 and the competition (defined by the 5% criterion for the free holoenzymes) starts for a lower number of alternative sigma factors, due to the sequestration of cores. In panels (v) and (vi), 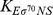 is smaller than 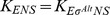 and the onset of competition is shifted to a larger number of 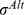 because the non-specific binding of 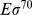 enhances the formation of housekeeping holoenzymes, so the competition is biased towards 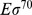. In the cell non-specific binding of the housekeeping holoenzyme and core are similar [46] and one can expect the non-specific binding of alternative sigma factors to be comparable as well. In that case, we can conclude form our results that the presence of non-specific DNA does not strongly affect sigma factor competition.

### Effect of transcript elongation

**Figure 5:**
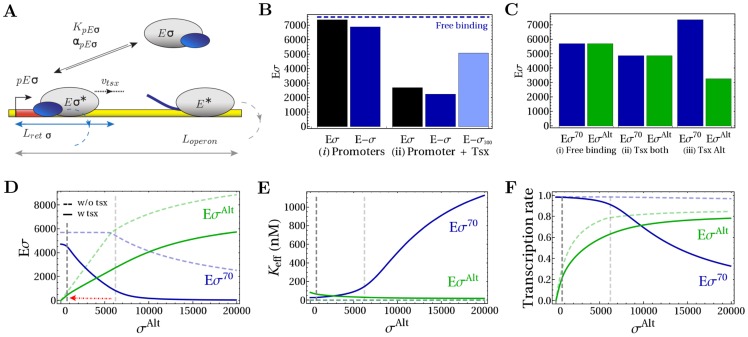
Effect of transcript elongation. (A) Active elongation sequesters core RNAPs for the length of the operon and sigma subunit for some nucleotides. (B) Formation of holoenzymes in the presence of one type of sigma factor without DNA (no specific binding and no transcription with 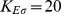 nM, dashed line), in the presence of specific binding (holoenzymes bind to promoter with 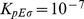 M but do not transcribe, case (i)) and in the presence of both specific binding and transcription (case (ii)). The black bars (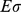) show the case when sigma factor and core unbind as holoenzyme (the binding affinity is described by the equilibrium dissociation constant), the dark blue (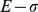) and the light blue bars (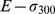) when sigma factor separates from core either after promoter unbinding or gene transcription and after 300 nucleotides, respectively (thus, the binding affinity is 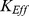). (C) Number of holoenzymes 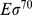 and 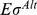 as a function of the copy number of alternative sigma factors in the absence of DNA (case (i)), with transcription of both *σ* ^70^- and *σ^Alt^*-dependent genes but with unbinding of sigma factor after 300 nucleotides and core at the end of the operon (case (ii)) and only with the transcription of the *σ^Alt^*-dependent genes (case (iii)). Values of the parameters are the same as in Figure 5B. (D) Formation of holoenzymes 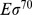 and 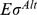 as a function of the copy number of alternative sigma factors without DNA (dashed lines) and transcript elongation (solid lines). (E) Modulation of the effective binding affinities 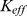 by sigma factor competition related to the case of Figure 5D. (F) Normalized transcription rate for *σ* ^70^- and *σ^Alt^*-dependent promoters as a function of the number of alternative sigma factors, related to the case of Figure 5D (with 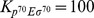 nM and 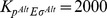 nM).

We next consider transcript elongation in more detail. When a holoenzyme binds to a specific promoter (Figure 5A), it starts to transcribe the associated genes with the initiation rate 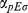. During early elongation, the sigma factor is typically released in a stochastic fashion [47]–[49], whereas the core RNAP is committed until it reaches a termination sequence. Thus, transcript elongation sequesters both core RNAPs and sigma factors, but for different amounts of time. The retention length of sigma was estimated to be between 100 [50] and 500 nucleotides [47]. With an elongation speed of 55 nt/sec, an average retention length of 300 nucleotides corresponds to a retention time of 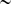 5 seconds. For comparison, core is sequestered for 30–120 seconds, assuming a range of operon lengths of 1500–6000 nucelotides.

In addition to sequestering those cores that are active in elongation, transcription also modulates the binding equilibrium between core and sigma, because the two are actively separated during early elongation. This modulation can be expressed by a binding equilibrium that is characterized by an effective dissociation constant
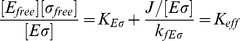
(2)with
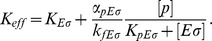



The two terms on the right hand side arise from the two pathways for the separation of sigma and core: the first term corresponds to the usual binding equilibrium where binding is balanced by unbinding, and the second term expresses active separation by transcription (see Methods). Here, 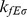 is the sigma-core binding rate (or the formation rate of the holoenzyme) and 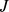 is the transcription rate per volume (initiations per second per volume), which effectively takes the place of a sigma-core dissociation rate (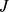 can be interpreted either as the transcription rate per volume of a specific gene in vitro or as an effective transcription rate of all active genes in the cell volume). In the second equality, we have expressed the transcription rate by the Michaelis-Menten model with the maximal transcription rate 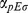 and the Michaelis constant 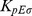 of the promoter. Equation 2 indicates that sigma-core dissociation constants measured in the presence of transcription, may not reflect the true binding strength, but rather a weaker effective affinity, because the initiation of transcription provides an additional pathway to dissociate core RNAP and sigma factor. If, however, the transcription rate is very low or if the transcribed sequence is short, i.e. shorter than or comparable to the sigma retention length, as it is often the case in *in vitro* assays, this effect can be neglected and one can use 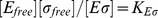 instead of Equation 2.

To disentangle the two effects of transcript elongation, sequestering of cores and modulation of sigma-core binding, we compare several scenarios for holoenzyme formation and promoter binding with a single sigma factor (Figure 5B). The blue dashed line shows the holoenzyme concentration in the absence of transcription (free binding, no promoters). Since binding between sigma and core is quite strong (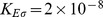 M), the number of holoenzymes is approximately given by the smaller one of the numbers of core RNAPs and sigma factors (here 7600 sigma factors and 11400 cores, see Equation 3 in Methods). Case (i) shows the number of free holoenzymes if holoenzymes can bind to promoters, but do not transcribe. When sigma factor and core RNAP are released together as a holoenzyme when unbinding from the promoter (black bar (i), 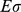), binding is simply characterized by the equilibrium dissociation constant 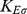. With 200 promoters, 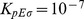 M and with the chosen parameters, (essentially) every promoter is occupied and the number of free holoenzymes is reduced by the number of promoters (which each sequesters one holoenzyme). When sigma factor and core RNAP are released as separate subunits when unbinding the promoter (blue bar (i), 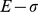), in addition to the sequestration, the binding between sigma and core is also modulated by the promoters, resulting in the weaker binding characterized by 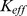 from Equation 2. As a consequence, the number of free holoenzymes is reduced more strongly than in the previous case. If we include transcript elongation, as shown in case (ii) in Figure 5B, RNAPs remain sequestered for a longer time, so the free holoenzyme concentration is reduced even more. We consider again the two instances, where core and sigma are released either as holoenzyme (black bar (ii), 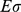, where we used 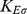) or separately at the end of the operon (blue 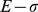 and light blue 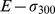 bars (ii), where we used 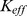). Here, in case (ii), the modulation of sigma-core binding plays a more prominent role. Indeed, when holoenzyme formation is limited by the availability of sigma factors, the sequestration of sigma factors by transcription reduces holoenzyme formation slightly (compare third and fourth bars). When, instead, the sigma factor is released after 300 nucleotides, the larger pool of free available sigma factors counteracts the weakening effect of 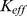 (light blue bar).

In the competition of two sigma factors, the transcription-dependent effective binding affinities can result in complex counterintuitive behavior. As an example, Figure 5C shows a scenario where transcription of housekeeping genes is abolished. The blue and green bars represent the housekeeping and alternative holoenzymes, respectively, which are characterized by the same parameters, 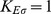 nM, 7600 sigma factors of each species and 11400 core RNAPs. The first two bars (case (i)) show the free binding of sigma factors and cores without transcription. Since the dissociation constant is small and sigma factors are in excess, cores are the limiting subunit and, due to the symmetry in the parameters, they are equally divided among the two species of sigma factors. The same happens in the presence of transcription, again with symmetric parameters, as shown by the second two bars (case (ii) with 200 promoters of each type, gene length of 2000 nucleotides, release of sigma factor and core after 300 nucleotides and at the end of the gene, respectively, and hence equal 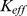 for both sigma factor species). The reduction with respect to the free binding case is given by sequestration by transcription and by the effect of 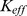. In case (iii), a shut-down of housekeeping genes frees a large number of core RNAPs, and thus one might expect that the production of all holoenzymes is stimulated. However, at the same time the binding between core and 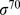 effectively becomes more tight, because it is no longer disrupted by the initiation of transcription. As a consequence, the formation of housekeeping holoenzyme is favored over the formation of alternative holoenzyme, resulting in the counterintuitive decrease of the concentration of the alternative holoenzymes. Note that the excess of sigma factors over core RNAPs allows the formation of more 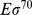 than in the free binding case without transcription. These predictions can be tested by multiple-round *in vitro* transcription experiments.

In Figure 5D, we show how transcript elongation affects sigma competition in the scenario of increasing concentration of alternative sigma factors. Here, the number of available cores that participate in the competition is effectively reduced by the number sequestered in transcript elongation with the effect that competition is expected to set in already for smaller sigma factors concentrations. In addition, the effective reduction in binding affinity between sigma and core smoothens the transition to the competition regime, further shifting the onset of competition to smaller sigma factor concentrations, as highlighted by the red arrow. The differential release of sigma factor and core is key to this shift: if sigma factors remained bound to core during elongation, the competition would be almost unaffected by the elongation process for a large range of parameters. The modulation of effective binding affinities 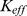 by the sigma factor competition during alternative sigma increase is shown in Figure 5E and the corresponding transcription rates, with a strong effect on the *σ* ^70^-dependent promoters, are shown in Figure 5F.

### Stringent response

Finally, we use our model to address the passive up-regulation of genes under the control of alternative sigma factors during the stringent response. The stringent response is a cellular program induced by amino acid starvation: shortage of amino acids leads to accumulation of uncharged tRNAs, which induces the synthesis of the signaling nucleotide ppGpp [51], [52]. ppGpp is a global regulator that directly or indirectly affects many processes, but its key regulatory role is to suppress the transcription of ribosomal RNA (rRNA) [53]. Since rRNA transcription accounts for up to 75 percent of all transcription in rapidly growing bacteria [15], [54], the *rrn* operons encoding the rRNAs sequester large numbers of RNAPs. These become free upon the stop of *rrn* transcription and thus become available to transcribe other genes. It has therefore been proposed that the stop or strong suppression of rRNA transcription passively up-regulates genes such as *σ* ^70^-dependent biosynthesis genes [55], [56] and alternative sigma factor-driven stress response genes [29], [32], [36]. A recent theoretical study has however estimated the effect on biosynthesis genes to be relatively small [15], so that direct activation of these genes by ppGpp (together with DksA) [57] is likely to be the dominant effect. The reason for the moderate effect is a relatively large pool of RNAPs non-specifically bound to DNA that buffers against such strong impact of the rRNA shut-down [15]. However, our results above indicate that non-specific binding does not affect the competition of sigma factors, so alternative sigma factor-controlled transcription may not be buffered against the release of core RNAPs from *rrn* operons. In the following, we therefore test the effects on sigma competition due to the stringent response within our model.

**Figure 6:**
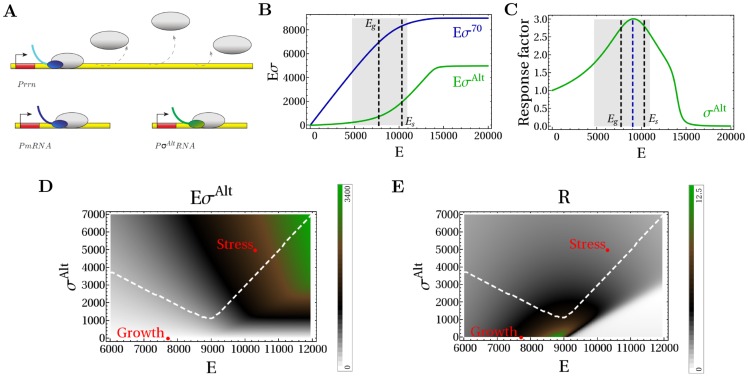
Stringent response. (A) During the stringent response RNA polymerases involved in rRNA transcription are quickly released to increase the pool of free cores. (B) Number of holoenzymes 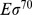 and 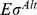 as a function of the copy number of core RNAPs. The black dashed lines show the number of available RNAPs during the exponential growth state (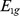) and during the stringent response state. The gray region shows the range of core RNAP for which there is sigma factor competition. (C) Response factor 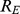 of the alternative sigma factor-dependent gene transcription (with 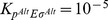 M) to an increase of concentration of RNAPs. The blue dashed line shows the maximal sensitivity, that for strong core-sigma binding, is found for 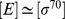 and lies in the competition region. (D) Number of alternative holoenzymes and (E) response factor 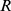 related to the *σ^Alt^*-dependent gene transcription as a function of the number of core RNAPs and alternative sigma factors (with 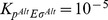 M). The white line encloses the region of sigma factor competition. The points show possible values of cores and alternative sigma factors for a cell in the exponential growth state and in the stringent state.

We first inspect the consequences of an increased concentration of core RNAPs due to their release from *rrn* operons (Figure 6A). We describe the total transcription in the cell by three classes of promoters: ribosomal RNA promoters (Prrn), *σ* ^70^-dependent mRNA promoters (PmRNA) and alternative sigma-driven promoters (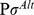
RNA). The stop of transcription of rRNA frees a large amount of cores (as well as some housekeeping sigma factors) that were sequestered there. For a simplified, but quantitative description of a bacterial cell during the stringent response, we have first to chose the parameters of the model: the numbers of cores and housekeeping and alternative sigma factors as well as the dissociation constants. We start from a previous description [15], based on the data of ref. [54] and consider *E. coli* cells growing with a growth rate of 2.5 dbl/h. Such a cell contains on average a total of 11400 RNAPs. Of these, approximately, 1100 are immature assembly intermediates, 2600 are transcribing rRNA and 700 are transcribing mRNA [15]. The remaining 7000 RNAPs are partitioned among non-specifically bound and free cores. We consider the immediate response to amino acid starvation, which is rapid and occurs on a timescale of 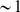 min. On this time scale, synthesis of new proteins is not expected to play an important role, so the total numbers of the molecular players can be considered as constant; in fact, the numbers of core RNAPs and 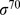 also do not change much in the transition from exponential growth to stationary phase [58], [59] (although their availability to form holoenzymes may be changed by sequestration, *e.g.* by anti-sigma factor and 6S RNA). Thus, the stop of *rrn* transcription releases 2600 core RNAPs, so that the total number of available cores to transcribe mRNA is increased to 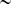 10300. The number of 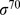 molecules per cell is less clear. While older studies have reported an excess of core RNAPs over 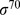 [59], [60], recently an 1.3–3-fold excess of the housekeeping sigma factor over core has been observed [38], [58], see also Table 1. However, the anti-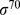 factor Rsd is also comparable in number to 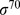 [58] and has a strong binding affinity for it [45]. Thus, it is likely that a substantial fraction of the housekeeping sigmas are sequestered by the anti-sigma factor. In the following, we use a plausible value of 9000 available (non-sequestered) 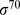 molecules per cell (see also Table 1). The main alternative sigma factors during the stringent response is 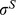 [61]. Below, we will consider a wide range of copy numbers of 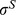, but for now we assume that there are 5000 copies present as estimated from observations during entry to stationary phase (60 percent of core [58], of which few are transcribing during growth). Finally, we use dissociation constants 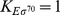 nM and 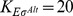 nM, consistent with experimental values as well as a Michaelis constant of 10 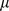
M for the binding of either holoenzymes to their cognate promoters. Mimicking the increase in core availability, we plot the numbers of holoenzymes of both types as functions of the number of core RNAPs in Figure 6B. Increasing core RNAP concentration allows the formation of holoenzymes until all sigma factors are engaged in holoenzymes. Competition between the sigma factors occurs in the range of core concentrations marked by the grey stripe. The upper limit of this stripe is given by the excess of sigma factors over cores and the lower limit depends on both the difference in sigma-core affinity and the 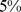 criterion (approximated by Equation S4 in the Text S1). The black dashed lines mark the numbers of available core RNAPs during exponential growth (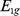) and after release of the *rrn*-transcribing cores in the stringent response (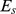), respectively. Here, both values lie in the region of competition. In the competition region, the number of alternative holoenzymes increases steeply, indicating that alternative sigma holoenzymes and, thus alternative sigma-driven transcription, is quite sensitive to the concentration of available core RNAPs. We quantify the sensitivity by determining a logarithmic response factor of the dependence of the transcription rate on the core concentration (see Equation 16 in Methods). A value of this parameter larger than one indicates hypersensitivity of the control. Indeed, in Figure 6C we find values up to 3, with the maximal sensitivity in the competition region. This result indicates that not only can alternative sigma-dependent transcription be induced passively by the stop of ribosomal RNA transcription, but also that even relatively small changes in core RNAP concentration are amplified into a pronounced increase of the transcription rate.

For a strong housekeeping sigma-core binding affinity, the response factor is larger than one as long as the number of cores is less than the total number of sigma factors (housekeeping and alternative) and the maximal sensitivity is found for 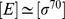 (blue dashed line in Figure 6C). If the number of housekeeping sigma factors (and hence the maximal sensitivity) lies between 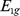 and 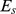, as in Figure 6C, or it is larger than both 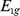 and 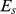, the *σ^Alt^*-dependent gene transcription is enhanced. On the contrary, if the number of 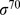 factors is smaller than the numbers of available cores during exponential growth and in the stringent response, hypersensitivity to increased core availability is lost, because the response factor can be in the region where sensitivity is smaller than unity. From this argument we can conclude that if housekeeping sigma factors are indeed in excess over core RNAPs, as suggested by some measurements [38], [58], strong amplification of passive up-regulation of *σ^Alt^*-dependent transcription can only be achieved if the housekeeping sigma factors are actively sequestered by some mechanism such as anti-sigma factors. We thus speculate that such thing may be a key function of the anti-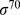 factor Rsd. If the latter condition is satisfied, our results indicate that an indirect (passive) up-regulation of the alternative sigma-dependent genes is possible, however such passive regulation requires that the system is tuned to work within or near the competition regime.

In addition to the release of core polymerases from the ribosomal genes, the response to stress such as amino acid starvation also involves the accumulation of alternative sigma factors via their increased synthesis and reduced degradation as well as through release of sigma factors sequestered by anti-sigma factors [21]. Hence, we now inspect the effect of a simultaneous increase in the concentrations of both core RNAPs and alternative sigma factors on the *σ^Alt^*-dependent transcription by repeating the analysis above for a wide range of 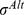 concentrations. Figures 6D and 6E show the concentration of alternative holoenzyme and the response factor as functions of the numbers of core RNAPs and alternative sigma factors. Estimated numbers of these molecules during exponential growth and in the stringent response are indicated by the red points. The white dashed lines in these Figures (for which Equation S3 in Text S1 provides a good analytical approximation) enclose the region of parameter values for which the system exhibits sigma factor competition. Thus, the stress response drives the cell into a state characterized by sigma factor competition. The formation of alternative holoenzymes is shown by the density plot of Figure 6D. It reaches its maximal level, which corresponds also to the maximal *σ^Alt^*-dependent transcription, for large numbers of core RNAPs and 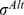 factors. One can see here that the number of 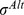 molecules has to exceed a threshold for competition to set. Thus for competition to set in upon the stop of *rrn* transcription, either a sizeable pool of 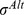 needs to be present already during exponential growth phase or the number of alternative sigma factors has to increase rapidly, *e.g.* by release from anti-sigma factors or by a stop of turnover. Once a level of 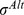 beyond the threshold indicated by the white line is reached, the contributions of increasing alternative sigma factor and core concentration to the increase in alternative holoenzyme formation and thus also *σ^Alt^*-dependent transcription are similar. The response factor of the transcription rate of *σ^Alt^*-dependent genes to an increase of core RNAPs is shown by the density plot of Figure 6E. This plot shows that the maximal response is achieved if the cell uses a small number of 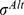 and if the amount of available cores is tuned to 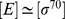. However, the formation of alternative holoenzymes remains very sensitive to changes in core availability in an extended range of the two parameters, which includes the estimated values for the exponential growth phase.

## Discussion

In this study, we have analyzed sigma factor competition in bacterial transcription, a mechanism by which gene can be controlled “passively” either through the cross-talk with another set of genes that are specifically regulated or by a change in the availability of the components of the transcription machinery, core RNAP and sigma factors. Extending previous work [38] we have developed a theoretical model that describes binding of sigma factors and core RNA polymerase, binding to promoters and transcription initiation and elongation, release of core and sigma factor as well as non-specific binding of the various molecular species to DNA.

We have used the model to describe several *in vitro* competition experiments [29], [30] that have determined effects of one sigma factor on the formation of holoenzymes involving a second sigma factor or the transcription rate of genes dependent on that second transcription factor. Very good agreement with the experimental data was obtained.

When competition between sigma factors is only for binding to core RNAP, the transcription of genes with saturated promoters are rather insensitive to such competition. Such promoters bind RNAP strongly, but initiate transcription at a relatively low rate so they are occupied by RNA polymerase most of the time [62] (called “poised” RNA polymerases in eukaryotic transcription [63]). The insensitivity against competition may be a mechanism for insulating the transcription of these genes from physiological perturbations related, *e.g.*, to stress responses, where sigma factor competition is induced. (It comes however at the price of a reduced dynamic range for regulation by transcription factors compared to unsaturated promoters [38]). If, however, promoters are recognized by two species of holoenzymes or promoters depending on different sigma factors overlap, even saturated promoters become affected by sigma factor competition.

The paradigmatic case of a saturated promoter in bacteria is a promoter under the control of 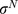 in *E. coli*. 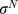 is structurally unrelated and mechanistically different from all other sigma factors in *E. coli*. Upon binding to a promoter, the *σ^N^*-holoenzyme stays in an inactive closed-complex state (poised state) and initiates transcription upon activation by an ATPase activator, which typically binds at distance from promoter and contacts the holoenzyme via DNA looping [64]–[67]. The kinetics of transcription initiation from a prototypical promoter of this class (P*gln* from *Salmonella typhimurium*) has recently been determined using single-molecule fluorescence [67] and from the resulting kinetic scheme, one can estimate that the promoter is indeed saturated at cellular concentrations of RNA polymerase and activator (Text S3). The transition to active elongation, which is the rate limiting step, is at least 10-fold smaller than the dissociation rate of the holoenzyme from the promoter (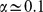 initiations per minute and 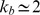 min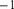, respectively). Thus even upon 10-fold activation, this promoter will remain close to saturation. As a consequence, our model predicts that this promoter should not be strongly affected by sigma factor competition. One can speculate that protection of *σ^N^*-driven transcription from competition may be related to the special role of 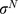, which despite being an alternative sigma factor also has housekeeping functions in controlling genes related to nitrogen metabolism [68]. We also note that not all *σ^N^*-driven promoters are saturated and protected from competition. Effects of sigma factor competition have been reported for several weaker 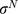 promoters (non-native to *E. coli*) [33], which thus should not be expected to be saturated according to our model. Interestingly, a series of hybrid promoters showed that the stronger-binding promoters (which according to our model should be closer to saturated) are less affected by the competition *in vitro* and *in vivo* [34], in agreement with the expectation from the model. It is also worth noting that the weak promoters for which competition was demonstrated were not native to *E. coli* and have specific non-housekeeping functions in their native hosts. In *E. coli* transcription from these promoters is induced during transition to stationary phase, similar to stress response transcription [32].

A key condition for competition is that sigma factors are in excess of core RNAP. When binding between sigma factors and core RNAP is strong, as experimentally observed with dissociation constants in the nM range and the competing sigma factors have approximately the same affinity for core, this condition is very intuitive: when core is in excess, all sigma factors are found in holoenzymes and no competition is obtained; competition sets in when the total number of available sigma factors is larger than the number of available core enzymes (not counting “unavailable” sigma factors and cores that are for example sequestered by anti-sigma factors or tied up in transcript elongation). This conditions is a general property of systems with one-to-one stoichiometry and competition for binding and similar observations have been made for small regulatory RNAs [6], protein sequestration [69] and proteases [7]. The competition gets more complex, when two sigma factors have different affinities for core. In that case, a stronger-binding sigma factor can start to displace a weak-binding sigma factor even without excess of total sigma factors. Measured sigma factor dissociation constants exhibit a clear hierarchy with the strongest binding for the housekeeping sigma factor [31]. However, there are some indications (although no definitive evidence) that the affinity of 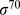 for core RNAP can be modulated by the alarmone ppGpp [29], [70], [71]. If this effect is specific to 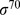 and not present for other sigma factors, it might modulate the sigma factor hierarchy and thereby enhance the competitive success of alternative sigma factors.

The hierarchy of sigma factor binding may also be affected by the transcriptional activity of the different holoenzymes, because transcription affects sigma factor competition in complex ways. Transcript elongation sequesters core RNAPs and, to a lesser extent, sigma factors, thus modulating the availability of these components. In addition, transcription also serves as a pathway for the effective dissociation of holoenzymes, effectively increasing their dissociation constant. While these effects are likely of minor importance *in vitro*, they should have a bigger impact *in vivo*, where there transcription does perturb the pool of free holoenzymes, thus calling into question how relevant the measured equilibrium dissociation constant are for the cell. These effects could be tested experimentally, *e.g.* by implementing the scheme of transcription studied in Figure 5C in multi-round *in vitro* transcription assays.

We have then studied the effects of non-specific binding of core RNAPs and holoenzymes to DNA. Non-specific binding may in principle interfere with sigma factor competition if different holoenzymes and/or core RNAP have different non-specific dissociation constants by shifting the binding equilibrium such as to minimize the overall binding energy. If however, these dissociation constants are approximately the same, as it is likely the case under physiological ionic strength [46], cytoplasmic and non-specifically bound components participate equally in sigma factor competition and the competition is independent of non-specific binding. As a consequence non-specific binding cannot buffer alternative sigma factor dependent transcription against passive effects due to the increased availability of core RNAPs during the stringent response. This conclusion is in contrast to earlier results for *σ* ^70^-dependent transcription of biosynthetic operons [15]. The two cases differ in the stage of the transcription initiation pathway in which they are subject to competition. Biosynthetic operon promoters compete with other genes and, more importantly, with non-specific binding sites on the DNA for the binding of holoenzymes. For alternative sigma dependent transcription, the competition occurs at an earlier stage, namely between the sigma factors binding to core, which is not affected by non-specific binding.

The last observation suggests that passive up-regulation of alternative sigma factor transcription can be expected during the stringent response, as proposed [34], [36]. In the stringent response, a global stress response to scarcity of amino acids, the transcription of ribosomal RNA is rapidly stopped. As ribosomal RNA transcription accounts for up to 75% of the total transcription in rapidly growing bacteria [54], [72], a large number of core RNAPs that were transcribing rRNA become available to transcribe other genes. Our calculations indicate that not only can this increase in core availability lead to a strong increase in the formation of alternative holoenzymes, and thus the concomitant transcription, but also that alternative sigma factor-dependent transcription may be hypersensitive to such changes in core availability.

We note that for a more detailed study of these passive effects *in vivo*, a consistent set of all concentrations of the different holoenzymes under different conditions (*e.g.* different growth rates or different time points during a stress response or entry to stationary phase) would be invaluable. One may even imagine a partitioning of sigma factors and holoenzymes similar to a recent study for core RNAP [16].

Finally, sigma factors have been proposed as versatile components for synthetic gene circuits. Sequestration of sigma factors by anti-sigma factors and competition for core RNAP provide mechanisms for genetic switches [43], [73], [74]. In this context, hyper-sensitive behavior may be a desired property of such switches and our theory could help to tune such systems into the required parameters regime. Recent experimental work in *B. subtilis* has demonstrated interesting pulsing dynamics of a sigma factor, driven by cycles of auto-activation and sequestration [44], [75]. Here we have only considered steady state situations, but our model can easily be extended to include such driving by coupling our description of the competition of sigma factors to a model for their synthesis.

## Methods

### Binding between sigma factor and core RNAP

Sigma-core binding is described by the equilibrium of the reaction
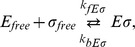
where 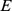, 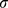, and 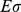 denote the core RNAP, the sigma subunit and the holoenzyme, respectively. The index “free” distinguishes the numbers or concentrations of free subunits that are not part of a holoenzyme from the total numbers or concentrations. The concentrations (denoted by 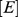, 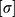, etc.) fulfill
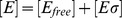


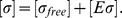



At equilibrium, they also fulfill
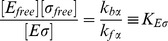
with the dissociation constant 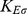. From these equations, the concentration of holoenzymes is found to be
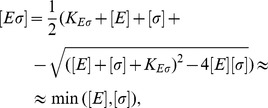
(3)where the approximation is valid for for very strong binding. Here 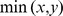 denotes the minimum function, which selects the smallest of its arguments 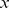 and 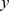.

If two sigma factors compete for core RNAPs, we have
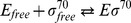


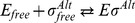
with the constraints
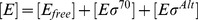
(4)

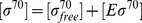
(5)

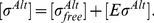
(6)


At equilibrium, the dissociation constants 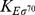 and 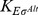 are given by
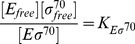
(7)

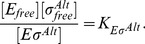
(8)


Analytical expressions for the holoenzyme concentration can be found for some special cases (see Text S1) but in general, these equations are solved numerically.

While the onset of sigma factor competition is abrupt for very strong sigma-core binding, in general, there is a smooth transition. Thus, we define the onset of competition to be the point where the alternative sigma factors cause a 5% reduction of 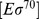 with respect to the situation without alternative sigma factors, *i.e.* for which
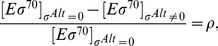
(9)where 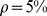. The onset of the competition is indicated by a grey dashed vertical line in the plots. In the limit of strong binding between core and sigma and for small 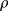, Definition 9 is equivalent to the condition 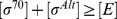 or equally to 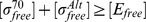.

The results above can be extended to the scenario where more than two sigma factor species (here generically 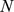) compete to bind to core RNAP. In this instance, the holoenzyme concentrations are obtained by solving
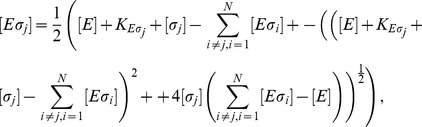
(10)where indexes 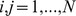 indicate the different sigma factor species. This yields the general form of Equation 1:
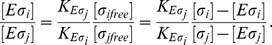



This expression shows that the ratio of concentrations of two kinds of holoenzymes depends only on the inverse of their relative dissociation constants, even if other species of sigma factors are involved in the competition.

### Transcription rate

The initiation of transcription process is described by a Michaelis-Menten model, so the rate of transcription of a gene (RNA synthesis rate per cell volume) with a promoter 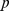 cognate to 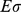 is
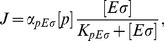
(11)where 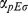 is the maximal initiation rate, 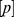 the concentration of the promoter and 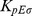 the Michaelis constant (which corresponds to the holoenzyme-promoter dissociation constant if binding equilibrates before the initiation of transcription). We usually plot the normalized transcription rate per gene (to which we refer simply as transcription rate), defined as
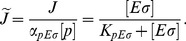
(12)


The case where a gene with a *σ* ^70^-dependent promoter (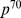) can be transcribed only by 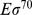, but binds also 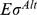, is a special case of repression at promoter level [1], and the transcription rate is given by
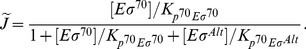
(13)


Here, the holoenzyme 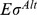 acts as a repressor with binding affinity 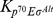 to the promoter 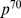.

The Michaelis-Menten model describes transcription initiation as consisting of two steps, binding of RNAP and initiation of elongation, while transcription initiation is known to proceed through several conformationally substeps including the formation of closed and open complexes, rounds of abortive initiation and promoter-proximal pauses [76]. However, these more complex schemes can generally be mapped to an effective Michaelis-Menten model with parameters that depend on the kinetic parameters of the more detailed scheme. We show this explicitly for the case of a *σ^N^*-controlled promoter in the Text S3.

### Anti-sigma factors

The binding of anti-sigma factor to the cognate sigma factor is described by the reaction
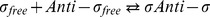
and the dissociation constant of the sigma-anti-sigma complex is given at equilibrium by
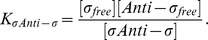



### Non-specific binding

Non-specific binding of core RNAP and holoenzymes to DNA (with binding sites 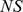) is described by the reactions
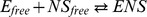


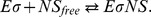



The number of free binding sites largely exceeds the number of occupied binding sites (specific and unspecific), hence 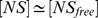 and
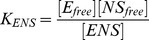


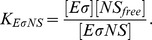



For the case of a single sigma factor species with 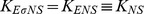, the holoenzyme concentration is given by
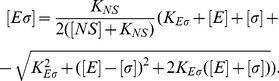
(14)


Dividing Equation 14 by Equation 3, we obtain the scaling factor 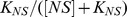 with respect to the free binding case. The same scaling factor is obtained for two sigma factor species, if 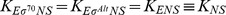.

### Transcript elongation

The binding of the holoenzyme to the cognate promoter 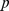 and the process of active transcription are described by the reactions
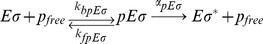


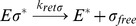


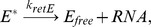
where 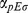 is the maximal initiation rate, starred quantities represent the units busy in active elongation with speed 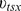 and committed for a retention length 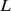. At steady state, we obtain
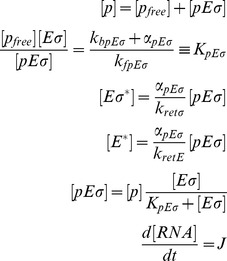
and the equilibrium dissociation constant is substituted by the effective dissociation constant
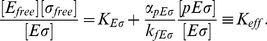
(15)


Equation 15 expresses the effective binding affinity due to the differential release of core and sigma during the active elongation. Sigma and core retention rates can be estimated from 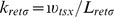 and 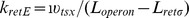, respectively. If core RNAP and sigma factor are released as a complex at the end of the operon, instead of the effective binding affinity of Equation 15, we obtain again the usual equilibrium dissociation constant 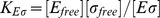.

### Response factor

The response coefficient 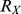 [77] characterizes the sensitivity of an observable (here, the normalized transcription rate of the *σ^Alt^*-dependent genes) to the change of a control parameter 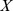 (here, either the total amount of core RNAPs or alternative sigma factors). The logarithmic response 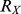 of the transcription rate to a change in 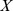 is
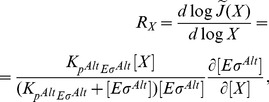
(16)where Equation 12 was used in the last expression. Since 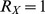 for 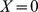 and 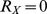 for 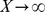, a necessary condition to have an absolute maximum of the response factor is 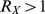. A value of the response coefficient larger than one denotes that the system is more sensitive to a change in the control parameter than a linear function. This instance is called hyper- or ultra-sensitivity. From Equation 16, 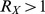 implies that the transcription rate is an increasing function of 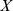, convex around its maximum 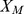. This maximum is found by solving
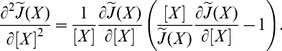
(17)


Generally, the maximum of the response factor 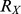 (and hence ultra-sensitivity) arises near the value where all 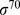 molecules are sequestered. From this point, free alternative sigma factors and cores are available to form alternative holoenzymes, inducing a steep increase in the number of 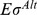 and eventually in the cognate transcription rate [78]. When the specific binding affinity 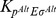 is strong, the system does not present any hyper-sensitivity. Thus, for a broad up-regulation of transcription, the corresponding promoter must be unsaturated.

From the analytical solutions of the free binding case with strong core-sigma binding affinities (Equations S2 in Text S1), we find that 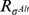 never has a maximum, whereas 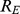, from Equation 17, has a maximum in the competition region around 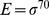, if the approximate condition
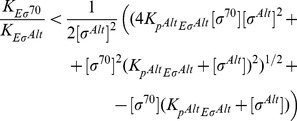
(18)is satisfied. In this case, 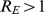 as long as 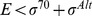. The right hand side of Equation 18 is always smaller or equal than one and for a small dissociation constant 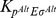, as we suppose to have in our simulations (see Table 1), it approaches one. Thus, a necessary (but not sufficient) condition for the presence of a maximum of 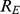 is 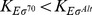.

The density plot of Figure 6E represents the value of 
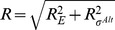
, which yields hypersensitivity for values larger than 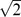.

## Supporting Information

Figure S1
**Mixed holoenzyme reconstitution experiment in the presence of all seven** ***E. coli*** **sigma factors.** An increasing equimolar amount of each sigma factor species was mixed with 400 nM of core RNAP and the concentration of holoenzymes of every species was registered (stars) [31]. We have fit these data with Equation 1 and have obtained the solid lines and the dissociation constants relative to 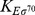 (Table S1). The index 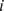 designates the different sigma factor species. Blue represents 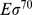, green 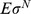, purple 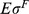, yellow 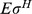, orange 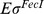, brown 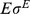, and cyan 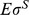.(TIF)

Figure S2
**Effect of anti-sigma factors.** (A) Formation of holoenzyme 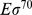 (blue lines) and 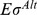 (green lines) as a function of the copy number of alternative sigma factors in the presence of a fixed amount of cores, housekeeping sigma factors and 5000 anti-alternative sigma factors. Here, the anti-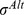 binds to the cognate sigma factor 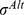 stronger than this latter to the core (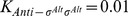 nM and 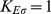 nM). The light dashed lines represent the case without anti-sigma factor, the grey lines the onset of competition and the red arrow highlight its shift. (B) Formation of holoenzymes as a function of the copy number of alternative sigma factors in the presence of a fixed amount of cores, housekeeping sigma factors and 19000 anti-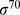. In this case, the anti-sigma factor binds to the housekeeping sigma factor weaker than this latter to the core (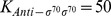 nM and 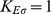 nM).(TIF)

Table S1
**Dissociation constants of different holoenzyme species relative to** 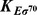 **, from reference** [31] **(second column) and according to our fit with** **Equation 1** **(third column).**
(PDF)

Text S1 **Analytical solutions.**
(PDF)

Text S2 **Values of the parameters used in the simulations.**
(PDF)

Text S3 **Estimate of association, dissociation and initiation rate from a** ***σ^N^*** **-dependent promoter.**
(PDF)
