# Supplementary figures and images for: A Model for Sigma Factor Competition in Bacterial Cells
Source: PLoS Comput Biol. 2014 Oct 9;10(10):e1003845. doi: 10.1371/journal.pcbi.1003845 (PMC4191881; doi:10.1371/journal.pcbi.1003845)

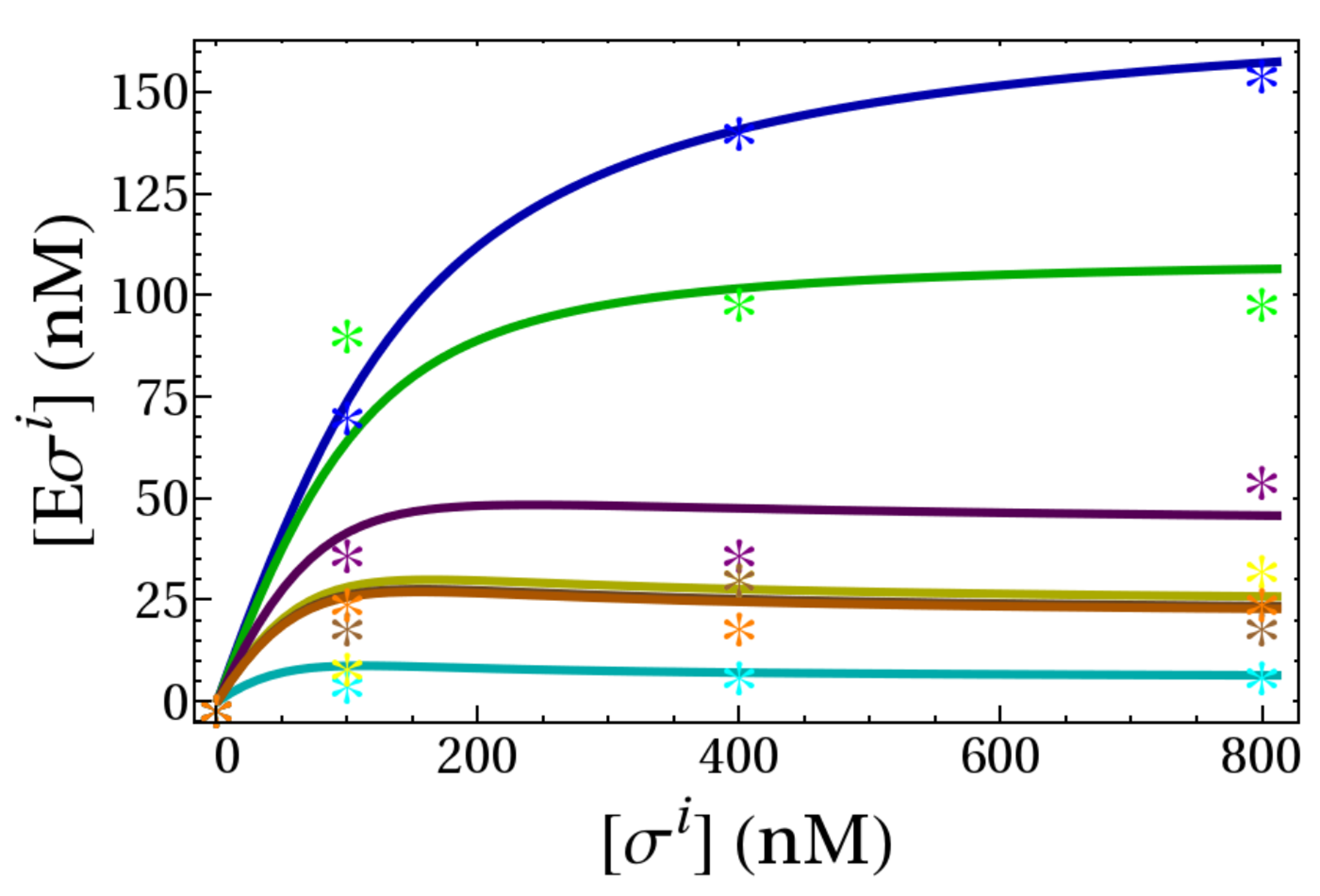

Supplement: Figure S1 — Mixed holoenzyme reconstitution experiment in the presence of all seven E. coli sigma factors. An increasing equimolar amount of each sigma factor species was mixed with 400 nM of core RNAP and the concentration of holoenzymes of every species was registered (stars) [31]. We have fit these data with Equation 1 and have obtained the solid lines and the dissociation constants relative to (Table S1). The index designates the different sigma factor species. Blue represents , green , purple , yellow , orange , brown , and cyan . (TIF) [file pcbi.1003845.s001.tif]

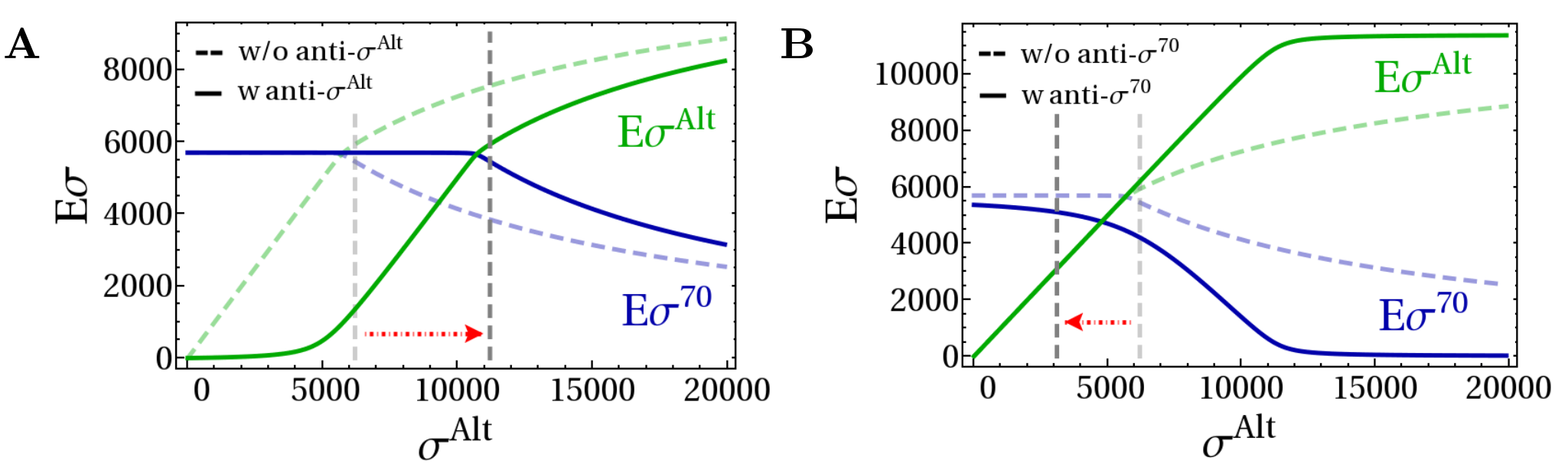

Supplement: Figure S2 — Effect of anti-sigma factors. (A) Formation of holoenzyme (blue lines) and (green lines) as a function of the copy number of alternative sigma factors in the presence of a fixed amount of cores, housekeeping sigma factors and 5000 anti-alternative sigma factors. Here, the anti- binds to the cognate sigma factor stronger than this latter to the core ( nM and nM). The light dashed lines represent the case without anti-sigma factor, the grey lines the onset of competition and the red arrow highlight its shift. (B) Formation of holoenzymes as a function of the copy number of alternative sigma factors in the presence of a fixed amount of cores, housekeeping sigma factors and 19000 anti-. In this case, the anti-sigma factor binds to the housekeeping sigma factor weaker than this latter to the core ( nM and nM). (TIF) [file pcbi.1003845.s002.tif]
