# Supplementary material for: A Model for Sigma Factor Competition in Bacterial Cells
Source: PLoS Comput Biol. 2014 Oct 9;10(10):e1003845. doi: 10.1371/journal.pcbi.1003845 (PMC4191881; doi:10.1371/journal.pcbi.1003845)
Supplement: Table S1 — Dissociation constants of different holoenzyme species relative to , from reference [31] (second column) and according to our fit with Equation 1 (third column). (PDF) [file pcbi.1003845.s003.pdf]

$\boldsymbol{\sigma^i}$	$\boldsymbol{K_{E\sigma^i}/K_{E\sigma^{70}}}$ [31]	$\boldsymbol{K_{E\sigma^i}/K_{E\sigma^{70}}}$ **our fit**
σ^N	1.55	1.59
σ^F	2.85	4.02
σ^H	4.75	7.29
σ^{FecI}	6.65	8.28
σ^E	9.35	8.05
σ^S	16.4	29.59

Table S1. Dissociation constants of different holoenzyme species relative to $K_{E\sigma^{70}}$, from reference [31] (second column) and according to our fit with Equation 1 (third column).
