# Supplementary material for: A Model for Sigma Factor Competition in Bacterial Cells
Source: PLoS Comput Biol. 2014 Oct 9;10(10):e1003845. doi: 10.1371/journal.pcbi.1003845 (PMC4191881; doi:10.1371/journal.pcbi.1003845)
Supplement: Text S1 — Analytical solutions. (PDF) [file pcbi.1003845.s004.pdf]

Text S1 - Analytical solutions

A general analytical solution for the problem of two competing sigma factor is not available, but different subsets of the parameters allow simplifications for which the model (as defined by Equations 4–8 in Methods) can be solved analytically. In the main text, we often use $K_{E\sigma^{70}} = K_{E\sigma^{Alt}} \equiv K_{E\sigma}$. In this case, the solution of the system for small core-sigma dissociation constants ($K_{E\sigma} \lesssim 10^{-6}$ M) is given by

$$[E\sigma^{70}] \approx \begin{cases} [\sigma^{70}] & [\sigma^{Alt}] \leq [E] - [\sigma^{70}] \\ \frac{[E][\sigma^{70}]}{[\sigma^{70}]+[\sigma^{Alt}]} & [\sigma^{Alt}] > [E] - [\sigma^{70}] \end{cases}$$
$$[E\sigma^{Alt}] \approx \begin{cases} [\sigma^{Alt}] & [\sigma^{Alt}] \leq [E] - [\sigma^{70}] \\ \frac{[E][\sigma^{Alt}]}{[\sigma^{70}]+[\sigma^{Alt}]} & [\sigma^{Alt}] > [E] - [\sigma^{70}] \end{cases} .$$

According to Equation 9 in Methods there is sigma factor competition when

$$[\sigma^{Alt}] \geq \begin{cases} [\sigma^{70}]\frac{\rho}{1-\rho} & [E] \leq [\sigma^{70}] \\ \frac{[E]-(1-\rho)[\sigma^{70}]}{1-\rho} & [E] > [\sigma^{70}] \end{cases} \tag{S1}$$

where ρ is the percentage threshold that we set at 5%. This condition was used to plot the gray dashed lines representing the onset of the competition in all figures where $K_{E\sigma^{70}} = K_{E\sigma^{Alt}}$. At single particle threshold ($\rho \to 0$), Inequality S1 reduces to $[\sigma^{Alt}] \geq 0$ when $[E] \leq [\sigma^{70}]$ and to $[\sigma^{Alt}] \geq [E] - [\sigma^{70}]$ when $[E] > [\sigma^{70}]$.

A second case of interest is the one in Figure 6 where $K_{E\sigma^{70}}/K_{E\sigma^{Alt}} \equiv K < 1$ and where both binding affinities are taken to be strong. Neglecting the pool of free holoenzymes in Equation 4 (see Methods), we obtain

$$[E\sigma^{70}] = \min\Bigg([E], [\sigma^{70}], \frac{1}{2(K-1)}\Big([E](K-1) - [\sigma^{70}] - K[\sigma^{Alt}] + $$
$$+ \sqrt{4[E](K-1)[\sigma^{70}] + ([E](1-K) + [\sigma^{70}] + K[\sigma^{Alt}])^2}\Big)\Bigg)$$
$$[E\sigma^{Alt}] = \min\Bigg([E], [\sigma^{Alt}], \frac{1}{2(K-1)}\Big([E](K-1) + [\sigma^{70}] + K[\sigma^{Alt}] + $$
$$- \sqrt{4[E](K-1)[\sigma^{70}] + ([E](1-K) + [\sigma^{70}] + K[\sigma^{Alt}])^2}\Big)\Bigg). \tag{S2}$$

According to Equation 9, sigma factor competition sets in when

$$[\sigma^{Alt}] \geq \frac{((1-\rho)m - [E])\big((1-K)(1-\rho)m - [\sigma^{70}]\big)}{K(1-\rho)m} \tag{S3}$$

where $m = \min([E], [\sigma^{70}])$. For $\rho = 5\%$ this expression agrees perfectly with the white dashed boundaries of Figures 6D and 6E. The curve has a minimum for $[E] = [\sigma^{70}]$. Solving Inequality

S3 with respect to $[E]$, we find that for $[\sigma^{Alt}]/[\sigma^{70}] \leq \rho(K(1-\rho)+\rho)/(K(1-\rho))$ there is no competition, for $\rho(K(1-\rho)+\rho)/(K(1-\rho)) < [\sigma^{Alt}]/[\sigma^{70}] \leq \rho/(K(1-\rho))$ there is sigma factor competition if

$$\frac{[\sigma^{70}]\rho - K[\sigma^{Alt}](1-\rho)}{(1-K)(1-\rho)\rho} \leq [E] \leq \frac{(1-\rho)(K([\sigma^{70}]+[\sigma^{Alt}]) - (K-1)\rho[\sigma^{70}])}{K(1-\rho)+\rho} \tag{S4}$$

and for $[\sigma^{Alt}]/[\sigma^{70}] \geq \rho/(K(1-\rho))$ if

$$[E] \leq \frac{(1-\rho)(K([\sigma^{70}]+[\sigma^{Alt}]) - (K-1)\rho[\sigma^{70}])}{K(1-\rho)+\rho}.$$

If $\rho \to 0$, the competition region reduces again to $[\sigma^{Alt}] \geq 0$ when $[E] \leq [\sigma^{70}]$ and to $[\sigma^{Alt}] \geq [E] - [\sigma^{70}]$ when $[E] > [\sigma^{70}]$. For $K < 1$, we know from the analysis of the response factor that R_E has a maximum at some value of $E > 0$ (see Methods). In fact, if the binding affinity between the alternative sigma factor and the core is much weaker than the corresponding housekeeping one ($K \ll 1$), the solution given by Equation S2 reduces to

$$[E\sigma^{70}] \approx \begin{cases} [E] & [E] \leq [\sigma^{70}] \\ [\sigma^{70}] & [E] > [\sigma^{70}] \end{cases}$$

$$[E\sigma^{Alt}] \approx \begin{cases} 0 & [E] \leq [\sigma^{70}] \\ [E] - [\sigma^{70}] & [\sigma^{70}] < [E] \leq [\sigma^{70}] + [\sigma^{Alt}] \\ [\sigma^{Alt}] & [E] > [\sigma^{70}] + [\sigma^{Alt}] \end{cases} .$$

According to Equation 16, $[E] = [\sigma^{70}]$ yields the maximal response factor, as marked by the dashed blue line in Figure 6C.
