# Supplementary material for: A Model for Sigma Factor Competition in Bacterial Cells
Source: PLoS Comput Biol. 2014 Oct 9;10(10):e1003845. doi: 10.1371/journal.pcbi.1003845 (PMC4191881; doi:10.1371/journal.pcbi.1003845)
Supplement: Text S2 — Values of the parameters used in the simulations. (PDF) [file pcbi.1003845.s005.pdf]

Text S2 - Values of the parameters used in the simulations

In the following, we discuss the experimentally determined values of the parameters of our model and how the values we adopted in our calculations (summarized in Table 1) were chosen. All parameter values are for *Escherichia coli.* Most of these parameters depend on the physiological condition of the cell and for some parameters, conflicting values have been reported by different labs or based on different experimental technique, so we have used a plausible range of values in our calculations. The values of Table 1 are approximation of *in vivo* or *in vitro* measurements. Since most numbers are growth rate dependent, we have assumed values similar to those that a cell has in the early stage of a stress response, after a previous period of rapid growth (here $\mu = 2.5$ dbl/h), a situation for which the maximal response is expected.

A. Average volume

We use an average cell volume of 1.32 fL, in accordance with the average volume given by reference [72] for a growth rate of 2.5 dbl/h. With this value, concentration of 1 nM corresponds to 0.8 molecules per cell or, alternatively, concentration of ~ 1.26 nM corresponds to one molecule per cell.

B. RNAPs and sigma factors

Recent investigations [38, 58] suggest that the total number of RNAP remains constant during the transition from exponential growth to stationary phases, but it still depends on the growth rate in the exponential phase. In Table I, we summarize different measurements for core RNAPs, housekeeping sigma factors and alternative sigma factors from three different labs. In our calculations, we used the value of 11400 RNAPs per average cell growing with a growth-rate of $\mu = 2.5$ dbl/h, as estimated by Bremer and Dennis [54]. A larger number has been reported by Grigorova *et al.* [38], which, however reflects the larger cell size for cells growing at 30°C compared to cells growing at 37°C.

The intracellular level of σ^{70} is believed to be higher than the amount of any individual alternative sigma species. According to recent measurements, which we summarize in Table I, housekeeping sigma factor is in excess over core RNAP (1.3 fold from Gross lab [38] and 3 fold from Busby lab [58]), while older measurements in the Ishihama lab [60] found core RNAP in 5-fold excess of σ^{70}. Concentration of housekeeping sigma seems to remain almost constant during the transition from exponential growth to stationary phase [60, 58] but to change with the growth rate [38]. To mimic the effects of σ^{70}-sequestration by anti-sigma factors, 6S RNA and elongating complexes, we set the number of housekeeping sigma factors to be half of the number of cores in our calculations. In our analysis of the stringent response, we estimate the number of housekeeping sigma factors from RNAP partitioning [15]. We assume that all the free RNAPs and the RNAPs non-specifically bound to DNA are housekeeping holoenzymes, plus a fraction of the transcribing RNAPs, which leads to approximately 9000 housekeeping sigma factors.

The concentration of alternative sigma factors depend on the cellular condition (see Table I). During exponential growth, housekeeping sigma factor is the most abundant. Even though during

stationary phase, the amounts of some alternative sigma factors are increased, the concentration of any single species does not exceed the concentration of total σ^{70} [38]. For example, numbers of σ^N and σ^F were found to be almost constant [60, 58]. By contrast, the concentration of other sigma factors such as σ^H and σ^S is known to increase considerably in altered physiological states [21]. From the data collected in Table I, we conclude that the total number of alternative sigma factors (the sum of all sigma species) can exceed the number of core polymerases. In our simulation we increase the concentration of the alternative sigma factor up to 20000 units either to mimic the shift from the exponential to the stationary phase or *in vitro* experiments with increasing alternative sigma factor concentration.

C. Dissociation constants

Sigma-core binding affinity varies with temperature and ionic conditions, as shown in Tables II–IV, where we collect values from the literature. A recent study *in vitro* [79] has systematically investigated these variations for σ^{70}, σ^H and σ^S and found not only the absolute values of the dissociation constants to vary with the conditios, but also the relative binding affinities for the different holoenzymes with $K_{E\sigma^{70}} < K_{E\sigma^S} < K_{E\sigma^H}$ at 20 °C, 150 mM NaCl; $K_{E\sigma^H} < K_{E\sigma^S} < K_{E\sigma^{70}}$ at 20 °C, 100 mM NaCl; $K_{E\sigma^{70}} < K_{E\sigma^S} < K_{E\sigma^H}$ at 30 − 35 °C, irrespective of NaCl concentration and $K_{E\sigma^S} < K_{E\sigma^H} < K_{E\sigma^{70}}$ at 40 °C.

Maeda *et al.* [31] reported a dissociation constant of 0.26 nM for the binding of σ^{70} and core RNAP. This value was obtained by fitting binding experiments with a Langmuir isotherm, implicitely assuming a constant concentration of sigma factors available for binding. We have fitted their data again using Equation 3 (which in contrast to the Langmuir expression accounts for the reduction of the concentration of free subunits by holoenzymes formation) and have obtained a dissociation constant of 0.02 nM, corresponding to about 10-fold stronger binding. In addition, Maeda *et al.* also performed a mixed holoenzyme reconstitution experiment with all seven sigma factors of *E. coli* (at 30 °C, 200 mM NaCl). They found that the hierarchy of the dissociation constants, measured using only the saturation condition, was $K_{E\sigma^{70}} < K_{E\sigma^N} < K_{E\sigma^F} < K_{E\sigma^H}, K_{E\sigma^{FecI}} < K_{E\sigma^E} < K_{E\sigma^S}$. Fitting the complete set of data with Equation 1, we obtained almost the same order: $K_{E\sigma^{70}} < K_{E\sigma^N} < K_{E\sigma^F} < K_{E\sigma^H} < K_{E\sigma^E} < K_{E\sigma^{FecI}} < K_{E\sigma^S}$ (Figure S1 and Table S1).

Since binding affinities between core and different sigma species are reported to be similarly strong at least *in vitro* [31], we often choose $K_{E\sigma^{70}} = K_{E\sigma^{Alt}}$. In addition we note that in most cases sigma-core binding is quite strong (nM dissociation constants, see Tables II–IV), which allows us to use approximations in the model such as neglecting free pools of the subunit that is limiting for holoenzyme formation .

In all scenarios, where transcript elongation is described explicitly, we also need the binding rate between sigma and core, as this enters the effective dissociation constants. This rate has not been measured, but a dissociation rate was measured in reference [80] for the σ^S holoenzyme and found to be around 10^{-3} sec^{-1}. Using this value and a dissociation constant of 1 nM, the holoenzyme formation rate $k_{fE\sigma}$ is obtained as 10^6 sec^{-1} M^{-1}. In all calculations, where the equilibrium constant is different from 1 nM, we kept the dissociation rate fixed.

D. Anti-sigma factors

In the cell, both anti-σ^{Alt} (such as FecR/σ^{FecI}, RseA/σ^{E}, FlgM/σ^{F}, RshA/σ^{H}) and anti-σ^{70} (such as Rsd or AsiA) can be present. They can vary in number from few molecules to large numbers. For example, RseA was measured to be present in cells at 200 and 400 molecules/cell during exponential growth and stationary phase, respectively. These values correspond to more than twice the amount of σ^{E}, to which RseA binds with a dissociation constant of approximately 100 nM [81].

Piper *et al.* [58] measured 3300 and 6200 anti-σ^{70} Rsd molecules per cell, during exponential growth and stationary phase, respectively, corresponding to 1.3-fold and 2.5-fold excess over the number of core RNAPs. For our simulations, we choose the amount of anti-σ^{70} to be 1.7-fold the amount of polymerases. Anti-σ^{70}-σ^{70} binding affinities were found to be relatively weak compared to the housekeeping sigma factor-core affinities: for example the complex AsiA-σ^{70} was measured to have a dissociation constant of 67 nM, and Rsd-σ^{70} 32 nM [45]. We assume a dissociation constant of 50 nM, in line with these estimates.

E. Non-specific binding

Every free DNA site can in principle be a non-specific binding site. *E. coli* has approximately 4.6×10^{6} base pairs per genome. Thus, with 3.8 genome equivalents per cell at a growth rate of 2.5 dbl/h [54], there are about 17.48×10^{6} non-specific binding sites per cell.

In reference [15], the dissociation constant for non-specific binding was estimated to be 3.1×10^{-3} M and in reference [1], 10^{-4} M. *In vitro* experiments [46] with 0.2 M NaCl or KCl found $K_{E\sigma NS} \simeq 10^{-5}$ M and $K_{ENS} \simeq 5 \times 10^{-7}$ M, and with ionic conditions that approximate the physiological conditions (in the presence of 0.01 M $MgCl_2$), the non-specific binding affinities of holoenzyme and core were found to be comparable ($K_{E\sigma NS} \simeq 3 \times 10^{-4}$ and $K_{ENS} \simeq 10^{-4}$). In our calculations, we used values for non-specific binding between 10^{-6} M and 10^{-2} M.

F. Specific binding to promoters and transcript elongation

In the *E. coli* genome, around 1800 promoters are under the control of the housekeeping sigma factor and 1300 depend on the alternative sigma factors [82]. However, only fractions of these promoters are active at any time. Also over a third are recognized by more than one holoenzyme species [82]. In addition, the promoter concentration varies during the life cycle of the cell with the replication of the genome and also with the growth conditions. Here, we used an average number of 200 active promoters/cell.

The length of the transcribed sequence is typically short in the *in vitro* experiments we analyzed, around 300 nucleotides. To describe *in vivo* situations, we choose an average length of 2000 nucleotides per operon and assume to have one promoter per operon that is recognized by a single holoenzyme species.

The promoters are characterized by the maximal transcription rate (α_p) and the Michaelis constant $K_{pE\sigma}$. For α_p, we take value similar to the *in vivo* values estimated in [83, 15]. $K_{pE\sigma}$ is taken to be comparable to the values reported affinities for *rrn* promoters and the *lac* promoter [72, 1].

The elongation speed of the transcribing RNA polymerase varies with growth conditions and depends on the transcribed sequence. Here we take an average transcription speed v_{tsx} of 55 nt sec^{-1}, as estimated for mRNA transcription in reference [54]. Based on the measurements of references [47, 50], we adopt a mean sigma factor retention length L_{ret} of 300 nucleotides.

Molecules/cell	Gross lab [38, 84]	Busby lab [58]	Ishihama lab [31, 59, 60, 85]
E	2600 ± 1300 $^{(1)}$	2598 ± 255 $^{(4)}$	3500 $^{(6)}$
	13000 ± 4000 $^{(2)}$	2574 ± 268 $^{(5)}$	8000 $^{(7)}$
σ^{70}	4700 ± 2400 $^{(1)}$	7283 ± 913 $^{(2)}$	$500 - 700$ $^{(8)}$
	17000 ± 4000 $^{(2)}$	7191 ± 898 $^{(5)}$	$500 - 700$ $^{(5)}$
σ^{Alt}	$\sigma^E = 3200 \pm 600$ $^{(1)}$		few σ^E
	$\sigma^E = 5500 \pm 1200$ $^{(2)}$		
	$\sigma^H = 20 \pm 5$ $^{(1)}$		few σ^H
	$\sigma^H = 120 \pm 34$ $^{(2)}$		
	$\sigma^H = 850$ $^{(3)}$		
		$\sigma^S < 1$ $^{(4)}$	$\sigma^S < 1$ $^{(8)}$
		$\sigma^S = 1615 \pm 383$ $^{(5)}$	$\sigma^S = 170 - 230$ $^{(5)}$
			110 σ^N, 30-350 σ^F, few σ^{FecI}

Table I: Average number of RNAP cores and sigma factors per cell from experimental measurements. (1): 0.45 dbl/h, 30 °C; (2): 1.33 dbl/h, 30 °C; (3): 6 min. after heat shock, 42 °C; (4): 1.6 dbl/h, 37 °C; (5): stationary phase, 37 °C; (6): 1 dbl/h; (7): 2 dbl/h; (8): growth phase, 37 °C.

$K_{E\sigma^{70}}$ (nM)	Conditions	Ref.
0.26	200 mM NaCl, ph 7.6, 30 °C, $^{(1)}$	[31]
3.3 ± 0.5	10 mM MgCl2, 100 mM Kglu, ph 8, 37 °C, $^{(1)}$	[86]
8	100 mM NaCl, ph 7.9, 22 °C, $^{(2)}$	[87]
50	250 mM NaCl, ph 7.9, 22 °C, $^{(2)}$	[87]
300	500 mM NaCl, ph 7.9, 22 °C, $^{(2)}$	[87]
3	100 mM Kglu, ph 7.9, 22 °C, $^{(2)}$	[87]
17	250 mM Kglu, ph 7.9, 22 °C, $^{(2)}$	[87]
21	500 mM Kglu, ph 7.9, 22 °C, $^{(2)}$	[87]
1.25 ± 0.22	10 mM NaCl, ph 7.4, 20 °C, $^{(3)}$	[79]
5 ± 0.66	10 mM NaCl, ph 7.4, 25 °C, $^{(3)}$	[79]
9.09 ± 0.71	10 mM NaCl, ph 7.4, 30 °C, $^{(3)}$	[79]
12.7 ± 1	10 mM NaCl, ph 7.4, 35 °C, $^{(3)}$	[79]
15.8 ± 1.7	10 mM NaCl, ph 7.4, 40 °C, $^{(3)}$	[79]
60 ± 10.4	150 mM NaCl, ph 7.4, 20 °C, $^{(4)}$	[79]
201 ± 23	150 mM NaCl, ph 7.4, 25 °C, $^{(4)}$	[79]
475 ± 23	150 mM NaCl, ph 7.4, 30 °C, $^{(4)}$	[79]
3000 ± 1700	150 mM NaCl, ph 7.4, 40 °C, $^{(4)}$	[79]
10.3 ± 1.1	100 mM NaCl, ph 7.4, 20 °C, $^{(4)}$	[79]
450 ± 29.2	100 mM NaCl, ph 7.4, 30 °C, $^{(4)}$	[79]
130	0.2 M KCl, ph 7.8, 30 °C, $^{(1)}$	From Table 2
21.1	50 mM KCl, 10 mM MgCl2, ph 7.5, 37 °C, $^{(1)}$	From Table 2

Table II: Collection of measured values of the σ^{70}-core dissociation constant $K_{E\sigma^{70}}$. (1) gel electrophoresis and/or filtration followed by staining or western blot and counts done through phosphor/fluor-imager; (2) Fret/Lret; (3) Langmuir - Blodgett trough; (4) surface plasmon resonance.

$\boldsymbol{K_{E\sigma^S}}$ **(nM)**	**Conditions**	**Ref.**
15.2 ± 3.7	10 mM MgCl2, 100 mM Kglu, ph 8, 37 °C, $^{(1)}$	[86]
68.2 ± 8.4	25 °C, $^{(4)}$	[80]
59 ± 3.9	10 mM NaCl, ph 7.4, 20 °C, $^{(3)}$	[79]
61.2 ± 4.9	10 mM NaCl, ph 7.4, 25 °C, $^{(3)}$	[79]
62.5 ± 5.6	10 mM NaCl, ph 7.4, 30 °C, $^{(3)}$	[79]
65.7 ± 4.6	10 mM NaCl, ph 7.4, 35 °C, $^{(3)}$	[79]
250 ± 22	150 mM NaCl, ph 7.4, 20 °C, $^{(4)}$	[79]
325 ± 23	150 mM NaCl, ph 7.4, 25 °C, $^{(4)}$	[79]
450 ± 22	150 mM NaCl, ph 7.4, 30 °C, $^{(4)}$	[79]
125 ± 11.3	100 mM NaCl, ph 7.4, 20 °C, $^{(4)}$	[79]
628 ± 32.2	100 mM NaCl, ph 7.4, 30 °C, $^{(4)}$	[79]

Table III: Collection of measured values of the σ^S-core dissociation constant $K_{E\sigma^S}$. (1)–(4) as in Table II.

$\boldsymbol{K_{E\sigma^H}}$ **(nM)**	**Conditions**	**Ref.**
0.8	100 mM NaCl, ph 7.9, 22 °C, $^{(2)}$	[87]
0.6	250 mM NaCl, ph 7.9, 22 °C, $^{(2)}$	[87]
2.4	500 mM NaCl, ph 7.9, 22 °C, $^{(2)}$	[87]
0.3	100 mM Kglu, ph 7.9, 22 °C, $^{(2)}$	[87]
1.3	250 mM Kglu, ph 7.9, 22 °C, $^{(2)}$	[87]
2	500 mM Kglu, ph 7.9, 22 °C, $^{(2)}$	[87]
22.5 ± 1.1	10 mM NaCl, ph 7.4, 20 °C, $^{(3)}$	[79]
57.1 ± 2.8	10 mM NaCl, ph 7.4, 25 °C, $^{(3)}$	[79]
72.9 ± 3.4	10 mM NaCl, ph 7.4, 30 °C, $^{(3)}$	[79]
92.9 ± 3.1	10 mM NaCl, ph 7.4, 35 °C, $^{(3)}$	[79]
125 ± 8	10 mM NaCl, ph 7.4, 40 °C, $^{(3)}$	[79]
103 ± 8	150 mM NaCl, ph 7.4, 20 °C, $^{(4)}$	[79]
312 ± 21	150 mM NaCl, ph 7.4, 25 °C, $^{(4)}$	[79]
614 ± 27	150 mM NaCl, ph 7.4, 30 °C, $^{(4)}$	[79]
1720 ± 140	150 mM NaCl, ph 7.4, 40 °C, $^{(4)}$	[79]
112 ± 9	100 mM NaCl, ph 7.4, 20 °C, $^{(4)}$	[79]
628 ± 32.2	100 mM NaCl, ph 7.4, 30 °C, $^{(4)}$	[79]
98.2	50 mM KCl, ph 7.5, 37 °C, $^{(1)}$	From Table 2

Table IV: Collection of measured values of the σ^H-core dissociation constant $K_{E\sigma^H}$. (1)–(4) as in Table II.

References

[79] Ganguly A, Chatterji D (2012) A Comparative Kinetic and Thermodynamic Perspective of the sigma-Competition Model in Escherichia coli. Biophys J 103: 1325–33.

[80] England P, Westblade L, Karimova G, Robbe-Saule V, Norel F, et al. (2008) Binding of the unorthodox transcription activator, Crl, to the components of the transcription machinery. J Biol Chem 283: 33455–64.

[81] Collinet B, Yuzawa H, Chen T, Herrera C, Missiakas D (2000) RseB binding to the periplasmic domain of RseA modulates the RseA:sigmaE interaction in the cytoplasm and the availability of sigmaE.RNA polymerase. J Biol Chem 275: 33898–904.

[82] Gama-Castro S, et al. (2011) RegulonDB version 7.0: transcriptional regulation of Escherichia coli K-12 integrated within genetic sensory response units (Gensor Units). Nucleic Acids Res 39: D98–105.

[83] Liang S, Bipatnath M, Xu Y, Chen S, Dennis P, et al. (1999) Activities of constitutive promoters in Escherichia coli. J Mol Biol 292: 19–37.

[84] Straus DB, Walter WA, Gross CA (1987) The heat shock response of E. coli is regulated by changes in the concentration of sigma 32. Nature 329: 348–51.

[85] Maeda H, Jishage M, Nomura T, Fujita N, Ishihama A (2000) Two extracytoplasmic function sigma subunits, sigma(E) and sigma(FecI), of Escherichia coli: promoter selectivity and intracellular levels. J Bacteriol 182: 1181–4.

[86] Colland F, Fujita N, Ishihama A, Kolb A (2002) The interaction between sigmaS, the stationary phase sigma factor, and the core enzyme of Escherichia coli RNA polymerase. Genes Cells 7: 233–47.

[87] Glaser B, Bergendahl V, Anthony L, Olson B, Burgess R (2009) Studying the salt dependence of the binding of sigma70 and sigma32 to core RNA polymerase using luminescence resonance energy transfer. PLoS One 4: e6490.
