# Supplementary material for: A Model for Sigma Factor Competition in Bacterial Cells
Source: PLoS Comput Biol. 2014 Oct 9;10(10):e1003845. doi: 10.1371/journal.pcbi.1003845 (PMC4191881; doi:10.1371/journal.pcbi.1003845)
Supplement: Text S3 — Estimate of association, dissociation and initiation rate from a σN -dependent promoter. (PDF) [file pcbi.1003845.s006.pdf]

Text S3 - Estimate of association, dissociation and initiation rate from a $\boldsymbol{\sigma}^N$-dependent promoter

In this section, we consider transcription initiation from σ^N-dependent promoters, a paradigmatic case of saturated promoters, in more detail. Friedman and Gelles [67] measured the kinetic steps involved in the initiation of transcription of the *S. typhimurium glnALG* promoter driven by the *E. coli* $E\sigma^N$ in a single molecule experiment with physiological concentrations of activators, and NTPs. They found the following kinetic scheme:

$$E\sigma^N + p_{free} \underset{k_{-1}}{\overset{k_1}{\rightleftharpoons}} pE\sigma_1^N \underset{k_{-2}}{\overset{k_2}{\rightleftharpoons}} pE\sigma_2^N \underset{k_{-3}}{\overset{k_3}{\rightleftharpoons}} pE\sigma_3^N \xrightarrow{k_4} p_{free} + E\sigma^{N*}, \tag{1}$$

where $pE\sigma_1^N$ and $pE\sigma_2^N$ are two (intermediate) closed complexes and $pE\sigma_3^N$ is the open complex. The rate k_3 describes the isomerization from the closed to the open complex and depends on the concentration of activators (NtrC in this specific case) and of ATP. The rate of transition from the open complex to elongation, k_4, depends on the concentration of NTPs. From the measured values of the rates at high activator and ATP concentration and at physiological concentration of NTPs ($k_{-1} = 0.32$ sec^{-1}, $k_1 = 2.1 \cdot 10^7$ M^{-1}sec^{-1}, $k_{-2} = 8 \cdot 10^{-3}$ sec^{-1}, $k_2 = 0.1$ sec^{-1}, $k_{-3} = 0.11 \cdot 10^{-3}$ sec^{-1}, $k_3 = 1.9 \cdot 10^{-3}$ sec^{-1} and $k_4 = 0.17$ sec^{-1}), k_3 is found to be the rate limiting step for the initiation of transcription.

In our model, the initiation of transcription is described by a Michaelis-Menten reaction scheme

$$E\sigma + p_{free} \underset{k_{fpE\sigma}}{\overset{k_{bpE\sigma}}{\rightleftharpoons}} pE\sigma \xrightarrow{\alpha_{pE\sigma}} E\sigma^* + p_{free},$$

which can be considered as a coarse-grained description of the complete scheme 1 above: The transcription rate obtained from Scheme 1 can be rewritten in Michaelis-Menten form

$$J = \alpha_{pE\sigma}[p]\frac{[E\sigma]}{K_{pE\sigma} + [E\sigma]} \quad \text{with} \quad K_{pE\sigma} = \frac{k_{bpE\sigma} + \alpha_{pE\sigma}}{k_{fpE\sigma}}$$

via the relations

$$\begin{aligned}
k_{fpE\sigma} &= k_1 \\
k_{bpE\sigma} &= \frac{k_{-1}k_{-2}k_{-3} + k_{-1}k_3k_4 + k_{-1}k_{-2}k_4}{k_{-2}k_{-3} + k_{-2}k_{-3} + k_2k_3 + k_{-2}k_4 + k_2k_4 + k_3k_4} \\
\alpha_{pE\sigma} &= \frac{k_2k_3k_4}{k_{-2}k_{-3} + k_{-2}k_{-3} + k_2k_3 + k_{-2}k_4 + k_2k_4 + k_3k_4}.
\end{aligned}$$

Note that $k_{bpE\sigma}$ and $\alpha_{pE\sigma}$ depend on the concentrations of activators, ATP, and NTPs via k_3 and k_4. Under conditions of high activator, ATP, and NTP concentration, these rates are obtained from the kinetics parameters measured by Friedman and Gelles as $k_{fpE\sigma} = 2.1 \cdot 10^7$ M^{-1}sec^{-1}, $k_{bpE\sigma} = 2.8 \cdot 10^{-2}$ sec^{-1}, $\alpha_{pE\sigma} = 1.7 \cdot 10^{-3}$ sec^{-1} and $K_{pE\sigma} = 1.4$ nM, from which $\alpha_{pE\sigma}$ (which is proportional to k_3 for small k_3) results as the rate limiting step. The transcription rate depends approximately linearly on k_3, but the Michaelis constant $K_{pE\sigma}$ of the *glnALG* promoter does not

change much upon a change in the concentrations of activators, ATP and NTPs. In particular, for a ten-fold down- or up-regulation of transcription (via modulation of k_3), $K_{pE\sigma}$ changes only 1.2-fold and 2.6-fold (*i.e.* $K_{pE\sigma} = 1.16$ nM and 3.7 nM), respectively. As a consequence, σ^N-dependent promoters of the type of the *glnALG* promoter are indeed saturated by holoenzymes, even for very low holoenzyme concentration (*e.g.* 10 nM which correspond to less than 10 molecules per cell). Thus, they can be expected to be insulated from sigma factor competition.
